# Multi-model analysis of gallbladder cancer reveals the role of OxLDL-absorbing neutrophils in promoting liver invasion

**DOI:** 10.1186/s40164-024-00521-7

**Published:** 2024-05-31

**Authors:** Dongning Rao, Jiaxin Li, Mao Zhang, Siyuan Huang, Lu Meng, Guohe Song, Jiaqiang Ma, Yingcheng Wu, Yifei Cheng, Shuyi Ji, Gaohua Wu, Lv Chen, Yuming Liu, Yang Shi, Jian Zhou, Fan Jia, Xiaoming Zhang, Ruibin Xi, Qiang Gao

**Affiliations:** 1 Department of Liver Surgery and Transplantation, Key Laboratory of Carcinogenesis and Cancer Invasion (Ministry of Education), Zhongshan Hospital, Liver Cancer Institute, Fudan University, Shanghai, 200032 China; 2 Peking-Tsinghua Center for Life Sciences, Academy for Advanced Interdisciplinary Studies, Peking University, Beijing, 100871 China; 3 Academy for Advanced Interdisciplinary Studies, Peking University, Beijing, 100871 China; 4 Shanghai Key Laboratory of Infectious Diseases and Biosafety Emergency Response, State Key Laboratory of Genetic Engineering, Institute of Infection and Health, National Medical Center for Infectious Diseases, Huashan Hospital, Fudan University, Shanghai, China; 5 Shanghai Institute of Immunity and Infection, Chinese Academy of Sciences, Shanghai, 200031 China; 6 BeiGene (Beijing) Co., Ltd, Beijing, China; 7 School of Mathematical Sciences, Center for Statistical Science, Peking University, Beijing, China; 8 Key Laboratory of Medical Epigenetics and Metabolism, Institutes of Biomedical Sciences, Fudan University, Shanghai, China; 9 State Key Laboratory of Genetic Engineering, Fudan University, Shanghai, China

**Keywords:** Gallbladder cancer, Metastasis, Tumor microenvironment, Neutrophils, Oxidized low-density lipoprotein, OLR1

## Abstract

**Background:**

Gallbladder cancer (GBC) is the most common and lethal malignancy of the biliary tract that lacks effective therapy. In many GBC cases, infiltration into adjacent organs or distant metastasis happened long before the diagnosis, especially the direct liver invasion, which is the most common and unfavorable way of spreading.

**Methods:**

Single-cell RNA sequencing (scRNA-seq), spatial transcriptomics (ST), proteomics, and multiplexed immunohistochemistry (mIHC) were performed on GBC across multiple tumor stages to characterize the tumor microenvironment (TME), focusing specifically on the preferential enrichment of neutrophils in GBC liver invasion (GBC-LI).

**Results:**

Multi-model Analysis reveals the immunosuppressive TME of GBC-LI that was characterized by the enrichment of neutrophils at the invasive front. We identified the context-dependent transcriptional states of neutrophils, with the Tumor-Modifying state being associated with oxidized low-density lipoprotein (oxLDL) metabolism. In vitro assays showed that the direct cell-cell contact between GBC cells and neutrophils led to the drastic increase in oxLDL uptake of neutrophils, which was primarily mediated by the elevated OLR1 on neutrophils. The oxLDL-absorbing neutrophils displayed a higher potential to promote tumor invasion while demonstrating lower cancer cytotoxicity. Finally, we identified a neutrophil-promoting niche at the invasive front of GBC-LI that constituted of KRT17^+^ GBC cells, neutrophils, and surrounding fibroblasts, which may help cultivate the oxLDL-absorbing neutrophils.

**Conclusions:**

Our study reveals the existence of a subset of pro-tumoral neutrophils with a unique ability to absorb oxLDL via OLR1, a phenomenon induced through cell-cell contact with KRT17^+^ GBC cells in GBC-LI.

**Supplementary Information:**

The online version contains supplementary material available at 10.1186/s40164-024-00521-7.

## Introduction

Gallbladder cancer (GBC) is the most common malignancy of the biliary tract and is particularly prevalent in South America, northern India and East Asia [13, 14]. As one of the most aggressive and lethal cancers, GBC lacks effective therapy, with an estimated 5-year overall survival of 5–13%. Among biliary tract cancers of different anatomic origins, patients with GBC have the least survival benefit when treating with conventional chemotherapy or investigational novel agents [15]. The standard of care for advanced GBC is cisplatin or gemcitabine, but the response to these chemotherapies is poor, which achieves a median overall survival of 11.7 months, underscoring the urgent need for novel therapeutic strategies. Next-generation sequencing studies have recently uncovered the genetic landscape and related pathway alterations in GBC, mainly involving pathways of TP53, ERBB family, KRAS, and PI3K [16]. However, so far, molecularly targeted therapy showed no advantages against the standard chemotherapy in GBC.

Gallstones present a major risk factor for GBC. While causality between gallstones and GBC is debated, most carcinogenesis of gallbladder happened in individuals with gallstones and follow the progressive changes in the gallbladder mucosa under chronic inflammatory conditions [14]. Once malignancy is established, tumor cells can recruit immune cells, further contributing to tumor progression through metastasis and invasion [17]. The integrative molecular analysis highlights the TME and immune profiles in gallbladder carcinogenesis and patient stratification [18, 19]. Of note, the ERBB pathway alterations prevalent in GBC contribute to the impaired patient survival through promoting immunosuppressive macrophage and regulatory T cell activation [20]. Thus, further in-depth characterization of the TME may provide novel biological underpinnings of GBC.

Involvement of neutrophils in tumor development have been noticed in many cancer types, including lung cancer, pancreatic cancer, liver cancer, colorectal cancer, breast cancer, and etc., with their dual roles in early and late stage of tumor progression, and particularly, their nonnegligible impact on tumor invasion and metastasis [21–26]. Additionally, neutrophils are major components of both chronic and cancer-elicited inflammation, which indicate their significantly impact on the initiation and progression of GBC [27, 28]. However, research on neutrophils in GBC remains limited. In this context, investigating the dynamics of neutrophils within the GBC microenvironment is essential for elucidating their role in disease advancement.

Moreover, GBC often exhibits infiltration into adjacent organs or distant metastasis, particularly liver invasion, which significantly impacts patient prognosis [14]. Therefore, elucidating the character of GBC liver invasion (GBC-LI) is imperative for developing effective therapeutic strategies. The state of art single-cell profiling with spatial information has depicted the TME reprogramming during the metastatic process in colorectal cancer liver metastasis [24, 29], breast cancer metastasis [30], and brain cancer metastasis [31]. In this study, by using single-cell RNA sequencing (scRNA-seq), spatial transcriptomics (ST), proteomics, and multiplexed immunohistochemistry (mIHC), we profiled multiple cell types including a relatively large population of neutrophils from GBC patients of different stages, allowing us to dissect the TME of GBC-LI and particularly, the dynamic cellular states of neutrophils at high resolution.

## Methods

### Patient samples and single-cell suspension preparation

Nineteen patients had surgery and were pathologically diagnosed as GBC from December 2019 to October 2021 were enrolled for scRNA-seq and ST. None of the patients received chemotherapy, radiotherapy, or any other anti- tumor therapy before surgery. This study was conducted in accordance with the ethical standards of the Research Ethics Committee of Zhongshan Hospital with patients’ informed consent. Written informed consent was obtained from all patients involved in this study for the use of their tissue samples and clinical information. In total, 38 samples were taken from 15 GBC patients of two subtypes for scRNA-seq: Localized GBC (GBC-Lo) with the tumor restricted in the gallbladder (*n* = 8) and GBC-LI with the tumor outgrowth of the gallbladder wall and directly invading the liver parenchyma (*n* = 7). Tumor, paired adjacent GB, and blood samples were taken from the patients. For GBC-LI patients, in addition to tumor regions within GB, we simultaneously obtained the tumor samples at the invasive front and the adjacent normal liver tissues. Tissue samples were obtained immediately following tumor resection, and then transported within RPMI-1640 medium with 10% fetal bovine serum on ice. Tissue samples were washed twice by cold 1× PBS (Gibco) and digested with Miltenyi Tumor Dissociation Kit and the GentleMACS (Miltenyi, Bergisch Glad- bach, Germany) following the manufacturer’s instructions. Blood samples were first washed by PBS and then went through the RBC lysis (Applygen Technologies, C1311). After centrifugation, the cell pellet was washed and re-suspended with MACS buffer (PBS containing 2% FBS). Before sorting, single-cell suspensions were stained with DRAQ5 (1:500, 10 min, CST, ) and DAPI (1:500, 2 min, Biolegend). For neutrophil sorting, samples were additionally stained with PE anti-CD66b (1:250, 10 min, Biolegend) before Live & Dead staining.

### Single-cell RNA sequencing

Libraries for scRNA-seq were generated using the Chromium Single Cell 3′ library and Gel Bead & Multiplex Kit from 10x Genomics (Genergy Bio-Technology, Shanghai). 10×Genomics Chromium barcoding system was used to construct a 10× barcoded cDNA library following the manufacturer’s instructions. All libraries were sequenced on Illumina HiSeq 4000 until sufficient saturation was reached.

### Preprocessing of the scRNA-seq data

CellRanger (v3.0.1) [1] was applied for read mapping and gene expression quantification. Cells with less than 250 genes or > 30% mitochondria genes were excluded. We also used three algorithms (DoubletFinder, DoubletDetection, and Scrublet) to find doublets and remove cells which were identified as a doublet by at least two algorithms. The total number of transcripts in each cell was normalized and followed by log transformation. Then we used Seurat (v3) to detect highly variable genes, perform PCA, graph-based clustering, t-SNE and UMAP.

### Annotation of immune cells

We first used SingleR [2] to classify cells into major cell types according to the Encode reference dataset, and based on expression of CD45/PTPRC, we divided immune cells and non-immune cells (epithelial cells, fibroblasts, endothelial cells). Then we applied the graph-based clustering method implemented in Seurat (v4.4.1) [3] to group immune cells into subtypes and each subtype was further annotated according to its marker genes (Additional file 1).

### Classification of malignant cells

As tumor cells harbor significantly more copy number variation (CNV) than normal cells, we estimated CNV from scRNA-seq following several steps. We first restricted our target cells to epithelial cells defined by SingleR and testified by commonly used marker for epithelial cells/cholangiocytes (EPCAM, KRT19). Then, the tumor cells were inferred by inferCNV [4], this was done by exploring the expression intensity of genes at different locations in the tumor genome using a set of normal cells (epithelial cells) as a reference (cutoff = 0.1, cluster_by_groups = T, denoise = T, HMM = T). In brief, genes were sorted according to their genomic location at each chromosome, and a sliding window of 100 genes was applied to calculate the average relative expression values to derive CNVi (CNV of the ith window). Next, we defined the CNV score of each cell as the mean of squared CNVi across all windows. Next, we defined the CNV score of each cell as the mean of squared CNVi across all windows. Malignant cells were then defined as those with CNV signal above 0.04.

### Differential expression and pathway analysis

Differentially expressed genes (fold change > 1.2 and *P* value < 0.001) were identified using FindMarkers with the bimod test implemented in Seurat (v4.4.1). Pathway enrichment analysis was performed using ClusterProfiler (v4.0.5) [5].

### The context-dependent scores

To delineate the transcriptional polarization of neutrophils across non-tumor and tumor environments, we compared their gene expression profiles from various sources (tumor, peritumor, and blood). Subsequently, we integrated differentially expressed genes (DEGs) specific to each sample type to define three context-dependent neutrophil activation scores: the Nontumor-Activating score (average expression of top 21 DEGs between peritumor and tumor intersected with differential genes between blood and tumor), the Tissue-Residing score (average expression of top 21 DEGs between peritumor and blood intersected with differential genes between tumor and blood), and the Tumor-Modifying score (average expression of top 21 DEGs between tumor and peritumor intersected with differential genes between tumor and blood).

### Developmental trajectory inference

Slingshot (v2.0.0) [6] implemented in dynverse [7] was applied to infer the developmental trajectory with the normalized expression values both in neutrophils and tumor cells.

### Gene regulatory network inference

Transcription factor activity was predicted by SCENIC (v1.2.4) [8], To reduce the computing time, a python implementation pySCENIC was used. GRNBoost2 [9] was used to determine correlation between transcription factors and other genes. Regulons were inferred based on publicly available motif binding databases provided by the Aerts lab. Finally, we employed Cytoscape to visualize the biologically significant target genes downstream of each transcriptional factor [10].

### Spatial transcriptomics

Fresh GBC tissues were washed and cut into 4–5-mm^3^ pieces. After cryosectioning, fixation, staining and brightfield imaging, on-slide tissue permeabilization, cDNA synthesis and probe release were performed. Finally, Visium spatial gene-expression library construction, and ST sequencing were conducted (Genergy Bio-Technology, Shanghai). Spaceranger-1.3.0 (v1.3.0) was applied for reads mapping and gene expression quantification. Then BayesSpace [11] was used to enhance the resolution of spatial transcriptome samples. BayesSpace achieves spatial clustering by modeling a low-dimensional representation of the gene expression matrix and making neighboring points belong to the same cluster via a spatial prior.

### Whole-exome sequencing and data processing

DNA was extracted from GBC tumor and non-tumor gallbladder tissues from these fifteen patients using a DNeasy Blood and Tissue kit (Qiagen), and DNA concentration and purity were determined using a NanoQuant Plate Infinite M200 PRO reader (Tecan Austria GmbH). After enrichment of exonic DNA fragments with a SureSelect Human All Exon Kit (Agilent, 50 Mb V5), sequencing was performed on Illumina NovaSeq 6000 (Novogene, Beijing). Raw sequencing reads were mapped to human genome version 38 (hg38) using BWA-MEM [12]. After removing duplicated reads, SNV and indel were detected using Mutect2 and annotated with funcotator.

### Mass spectrometry

Neutrophils were first isolated from peripheral blood, liver, and tumor samples of GBC-LI patients and blood of healthy donors. Around 1⋅10^4^ cells in each sample were prepared for protein extraction and digestion for mass spectrometry (APTBIO, Shanghai). Data Independent Acquisition (DIA) was performed on a timsTOF Pro mass spectrometry (Bruker) that was coupled to Nanoelute (Bruker) liquid chromatography, then the DIA data was analyzed with SpectronautTM 14.4.200727.47784 searching the database.

### Multiplexed immunohistochemistry

First, 4 μm FFPE sections of GBC tumor were deparaffinized in xylene and then rehydrated in 100%, 90%, 80% and 75% alcohol. Then antigen unmasking was processed in a near boiling epitope retrieval solution (100X citrate buffer, pH 6.0 or 50X EDTA buffer, pH 9.0) for 10 min, and endogenous peroxidase was inactivated by incubating with 3% H2O2 for 20 min at room temperature. After washed with tris buffer, sections the sections were incubated with 10% normal goat serum and then incubated with the primary antibodies for overnight at 4 °C–4 h at room temperature. Next, sections were incubated with the corresponding HRP-conjugated secondary antibodies (MP-7451 and MP-7452, VectorLab) for 30 min at room temperature. Then sections were incubated with Opal-520, Opal-540, Opal-570, Opal-620, Opal-650, or Opal-690 (PerkinElmer) for visualization of each antibody. DAPI were added at last for detecting the cell core. Finally, the images of sections were taken by Vectra and processed by Inform. The following antibodies were used: anti-CD66b (1:1500, abcam, ab197678), anti-PanCK (1:1000, abcam, ab7753), anti-CD68 (1:1000, abcam, ab955), anti-FOXP3 (1:500, abcam, ab20034), anti-LGALS3 (1:500, Proteintech, 14979-1-AP), anti-oxLDL (1:500, Abcam, ab14519), anti-KRT17 (1:500, Proteintech, 17516-1-AP), OLR1 (1:500, Proteintech, 11837-1-AP).

### Flow cytometry and cell sorting

Before staining, single cell suspensions were first incubated with Fc receptor blocking on ice for 10 min. Then cells were stained with Live & Dead stain (1:1000) for 5 min at room temperature. After staining cells were washed and re-suspended with FACS buffer (PBS containing 2% FBS and 0.1% sodium azide). Surface markers staining were also processed in FACS buffer for 15 min at room temperature. For intracellular staining, cells were fixed with Fixation/Permeabilization buffer for 1 h at 4 °C and washed with 1⋅ Permeabilization buffer, then labeled with the Rabbit primary antibody for 1 h at 4 °C. The cells were then labeled with Goat anti-Rabbit IgG (H + L) Cross-Adsorbed Secondary Antibody, FITC at a dilution of 1:500 for 1 h at 4 °C. Finally, cells were acquired by BD LSR Fortessa and analyzed by Flowjo. For cell sorting, cells were processed by BD FACS Aria II after surface stain. The following antibodies against Human were used: APC anti-CD66b (1:500, Biolegend, 396,905), BV510 anti-CD33 (1:250, Biolegend, 366,609), FITC anti-CD3 (1:100, BioLegend, 317,305), anti-SREBF1 (1:100, Proteintech, 14088-1-AP), PE anti-LOX1 (1:500, BioLegend, 358,603), anti-KRT17 (1:500, Proteintech, 17516-1-AP).

### Co-culture of neutrophils and cancer cell lines

Immune cells were isolated from peripheral blood from healthy donors and cancer patients respectively. Blood samples were first diluted by PBS, neutrophils and PBMCs were isolated by Ficoll gradient centrifugation and RBC lysis. Each main immune lineage will be distinguished by surface staining after the cholesterol uptake assay. GBC cell lines (EH-GB1, GBC-SD, and NOZ) and hepatocellular cancer cell lines (Huh7 and MHCC97-H) were seeded in 48-well plate with 10⋅10^5^ cells/well overnight. Neutrophils and PBMCs were monocultured or co-cultured with tumor cells for 30 min at 37 °C for further cholesterol uptake assay.

### Cholesterol uptake assay

For measuring oxLDL or LDL uptake, cells were incubated with DMEM containing 50ug/ml Dil-oxLDL or Dil-LDL for 30 min at 37 °C. After incubation, cells were washed with MACS buffer and harvested for further surface and intracellular staining as described above. Eventually, cells labled with Dil-oxLDL or Dil-LDL will be detected by flow cytometry. For microscpic detection of oxLDL uptake, tumor cells were first stained with 10 μm Calcein-AM for 30 min, then washed with PBS and co-cultured with neutrophils (Tumor: Neu = 1:1) for 30 min. For experienments involving cholesterol receptors inhibition, neutrolization antibodies were added after the tumor-neutrophil co-culture and then incubated for 30 min before Dil-oxLDL supplement. To acquire the oxLDL-absorbing neutrophils for subsequence analysis, we utilized FACS to isolate Dil-oxLDL^high^ neutrophils from the GBC-neutrophil co-culture system. Human Dil-oxLDL and Dil-LDL were obtained from Yiyuan Biotechnologies (Guangzhou, China).

### Transwell invasion assay

Transwell invasion assay was performed using 24 Cell Culture Insert Plate (LABSELECT, 14,341). DMEM containing Matrigel (Corning, BD Biocoat, 356,234) of 1:8 were coated in each insert for 3 h. Bottom chambers were seeded with 4⋅10^5^ of EH-GB1 cells, or 2⋅10^5^ EH-GB1 cells co-cultured with 2⋅10^5^ neutrophils. Cells in the bottom chambers were incubated in DMEM with 10% FBS, with or without 50ug/ml oxLDL. Then 3⋅10^4^ of EH-GB1 cells were seeded on the insert of each top chamber within DMEM. After 12 h of culture in 37 °C, the cells on the membrane were fixed by 4% methanol and then stained with 1% crystal violet. The invaded tumor cells to the lower surface was photographed by PerkinElmer Vectra 3 and counted in ImageJ2 (2.3.0).

### Cytotoxity assay

EH-GB1 was seeded in 48-well palte at 5⋅10^5^ cells/well for overnight. For the viability assay, EH-GB1 was first stained with 10 μm Calcein-AM for 30 min. After washing with PBS, tumor cells were co-cultured with neutrophils (Tumor: Neu = 1:3) and with or without 50ug/ml oxLDL (Yiyuan Biotechnologies) addition at 37 °C for 1 h or 24 h. Finally, the tumor cells were digested and collected for flow cytometry. For the apoptotic assay, EH-GB1 cells were directly co-cultured with neutrophils or oxLDL-absorbing neutrophils purified by FACS and collected for Annexin V-FITC (BD 556,547) staining after 24 h.

### Gene expression analysis using qRT-PCR

qRT-PCR was performed to detect the gene expression of neutrophils from different tissues of GBC patients, neutrophils of different in vitro stimulation, and EH-GB1 cells with siRNA knockdown of multiple genes. qRT-PCR was performed using 5 ng of cDNA per each well according to the manufacturer’s instructions. The following primers were used: S100A12 (F, AGCATCTGGAGGGAATTGTCA; R, GCAATGGCTACCAGGGATATGAA), FCN1 (F, GGCAGGTGTCATTGGAGAGAG; R, GTCGCACACGACTGAGACTG), IL1B (F, ATGATGGCTTATTACAGTGGCAA; R, GTCGGAGATTCGTAGCTGGA), CXCL8 (F, TTTTGCCAAGGAGTGCTAAAGA; R, AACCCTCTGCACCCAGTTTTC), PI3 (F, CACGGGAGTTCCTGTTAAAGG; R, TCTTTCAAGCAGCGGTTAGGG), CTSD (F, TGCTCAAGAACTACATGGACGC; R, CGAAGACGACTGTGAAGCACT), SREBF1 (F, GAAGATGTACCCGTCCATGCCC; R, GCTTCTCCGCATCTACGACCAG), OLR1 (F, TTGTTCAGCTCCTTGTCCGCAA; R, TCTGGGCTCTCATGTTTGGCAC), SQLE (F, CTCATCTGAGGTCCATGCCAGC; R, AGCACCACTACTGAGAAGGGCT), LDLR (F, CGAAGATGGCTCGGATGAGTGG; R, TATCTTCGCATCTTCGCTGGGC), SREBF2 (F, CCCCTGGGCCAGAAGTTTTTCA; R, GACGTTGAGGCTGCTCCATAGG), NPC1L1 (F, TACTTGGGTATCCGCTCCTCCC; R, CGCTGATGTGGCACATGGAGTA), CD36 (F, CTCTTTCCTGCAGCCCAATGGT; R, TGGGTTTTCAACTGGAGAGGCA), KRT17 (F, GCACCAAGUUUGAGACAGAGC; R, UCUGUCUCAAACUUGGUGCGG), ANXA1 (F, GCAGAGUGUUUCAGAAAUACA; R, UAUUUCUGAAACACUCUGCGA), IGFBP7 (F, CGAGCAAGGUCCUUCCAUAGU; R, UAUGGAAGGACCUUGCUCGCA), CD9 (F, GGAUGAGGUGAUUAAGGAAGU; R, UUCCUUAAUCACCUCAUCCUU).

### Gene silencing

Gene silencing was accomplished by siRNAs targeting KRT17 (F, 5’- GCACCAAGUUUGAGACAGAGC − 3’; R, 5’- UCUGUCUCAAACUUGGUGCGG − 3’), ANXA1 (F, 5’- GGUUAAAGGUGUGGAUGAAGC − 3’; R, 5’- UUCAUCCACACCUUUAACCAU − 3’), IGFBP7 (F, 5’- CGAGCAAGGUCCUUCCAUAGU − 3’; R, 5’- UAUGGAAGGACCUUGCUCGCA − 3’), and CD9 (F, 5’- GGAUGAGGUGAUUAAGGAAGU − 3’; R, 5’- UUCCUUAAUCACCUCAUCCUU − 3’).

### Western blotting

EH-GB1 cells with siRNA knockdown of KRT17 were lysed in lysis buffer (50 mM Tris (pH 7.4), 150 mM NaCl, 1% Triton X-100, and 1mM EDTA (pH 8.0) supplemented with protease inhibitor Roche cOmplete™ Mini and 1mM PMSF, 1 mM Na3VO4, and 1 mM NaF for 30 min on ice, and cell debris was removed by centrifugation at 13,000 rpm for 30 min. After being boiled at 95 °C for 5 min, the samples were separated by 10% SDS-PAGE and were transferred to a PVDF membrane and then probed with anti-KRT17 (1:5000, Proteintech, 17516-1-AP) and anti-β-actin (1:10000, CST, Catalog#4967) antibodies respectively. Then, the membranes were immunoblotted with anti-rabbit IgG secondary antibody (1:1000, Proteintech, RGAR001). Western blotting images were captured by a Tanon-5200 Chemiluminescent Imaging System (Tanon).

### Statistical analysis

All statistical analyses were performed using the R software (v. 4.1.1) and the GraphPad Prism (v.8.0.0). All the R code used in the analysis described in this study is provided in Additional file 2. Unless otherwise stated, statistical significance was estimated by two-tailed unpaired t-test, one-way ANOVA with Tukey’s multiple comparisons test or two-way ANOVA with Sidak’s multiple comparisons test, with *P* < 0.05 being considered statistically significant. Survival was analyzed with Kaplan–Meier survival estimates and log-rank tests. The ward. D2 method were used to perform unsupervised hierarchical clustering, and the emerging subgroups of patients were used for survival analysis.

## Results

### An immunosuppressive environment enriched with neutrophils in GBC liver invasion

**Fig. 1:**
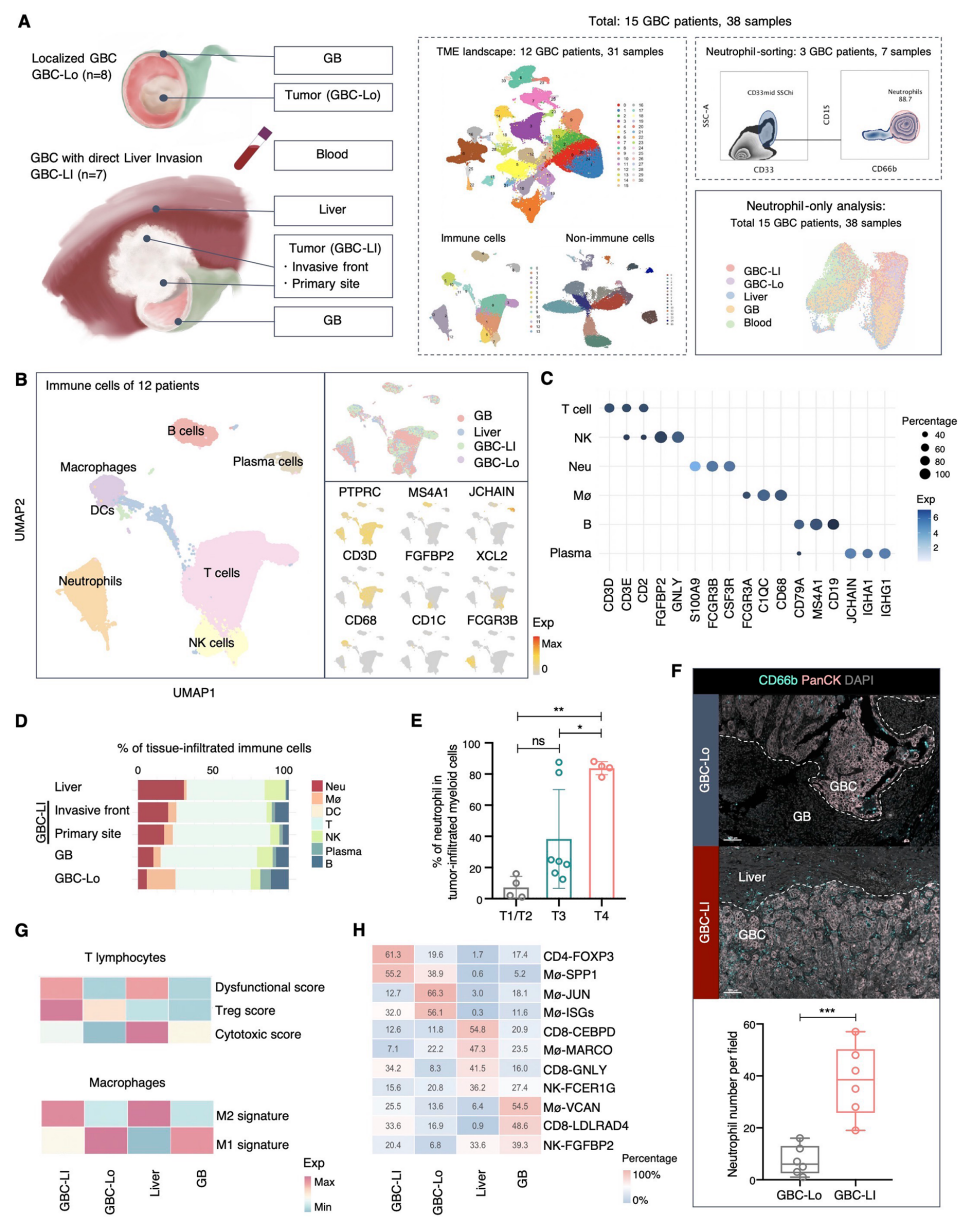
An immunosuppressive environment enriched with neutrophils in GBC liver invasion. (**A**) Schematic overview of the scRNA analysis workflow. (**B**) UMAP of immune cells from GBC patients colored by cell types (left) and sample origin (top right), and the expression of representative genes (bottom right). (**C**) DotPlot represent the expression level and percentage of the top differentially expressed genes in each type of immune cells. (**D**) The proportion of each immune cell type among different tissues. (**E**) The proportion of neutrophils in tumor-infiltrated myeloid cells among GBC tumor samples of different T stages according to the tumor, lymph node, metastasis (TNM) staging (The Eighth Edition AJCC Cancer Staging). Statistical analyses were performed using one-way ANOVA with Tukey’s multiple comparisons test. (**F**) Representative mIHC images and quantification of the CD66b^+^ neutrophils on the tissue sections of GBC-Lo and GBC-LI. GBC cells were stained with PanCK. The dash line separated the tumor and non-tumor area. Scale bars, 100 μm. CD66b^+^ neutrophils were counted in different GBC regions at 20x field-of-view. Comparison was performed using two-tailed unpaired t-test. (**G**) Heatmap showing the top immune subsets enriched in each sample type and their percentage in according to cell types from each sample type. in each sample. (**H**) Heatmap of functional scores for T lymphocytes (top) and macrophages (bottom) Data are presented as mean with SD. *P < 0.05, **P < 0.01, ***P < 0.001, ****P < 0.0001 See also Figure S1

To understand TME of GBC with direct liver invasion, we obtained multi-region samples from GBC patients for scRNA-seq. In total, 38 samples of tumor, paired adjacent normal tissues, and blood samples were taken from 15 GBC patients of two subtypes: GBC-LI and Localized GBC (GBC-Lo) (Figs. 1A and S1A, Table S1, Methods). Single cells were isolated from 5 types of samples to generate the TME landscape of GBC. Additionally, tissue sections of tumor-liver boundary from 4 independent GBC-LI patients were sequenced by ST (Methods). After quality control and doublets removal, a total of 273,059 high-quality cells were retained for further analysis. Graph-based clustering analysis and cell type annotation revealed 7 major immune cell types: T cells (CD3D), NK cells (FGFBP2 and GNLY), B cells (MS4A1), plasma cells (JCHAIN), macrophages (CD68), dendritic cells (CD1C and LILRB4), and neutrophils (FCGR3B and S100A9) (Figs. 1B, 1C, and S1B, Table S2, and Additional file 1). Non-Immune populations were classified into fibroblasts (PDGFRA), endothelial cells (PECAM1), and normal/malignant epithelial cells (EPCAM and KRT19). The major cell types we identified were largely consistent with the previous scRNA-seq data of GBC [20] and other hepatobiliary cancers [32–34].

The immune composition varied across different sample types. In particular, neutrophils had a relatively high infiltration rate (∼ 25% of all immune cells) in GBC-LI (invasive front) as well as in the paired adjacent livers, while exhibiting less abundance (∼ 5%) in GBC-Lo and GBs (Fig. 1D). The tumor invasive front had a higher proportion of neutrophils than the paired intratumor sites of GBC-LI, indicating the potential role of neutrophils in facilitating the liver invasion of GBC. Among the tumor-infiltrated myeloid cells, we discovered that the percentage of neutrophil in myeloid cells was significantly higher in GBC of T4 stage (TNM staging system) than in other earlier stages, further associating the neutrophil infiltration with the invasiveness of GBC (Fig. 1E). MIHC staining on GBC sections confirmed the preferential enrichment of neutrophils in GBC-LI versus GBC-Lo and further revealed their distribution at the invasive margin (Fig. 1F). We applied neutrophil signature inferred from scRNA-seq data on the ST plots, again confirming the relatively high density of neutrophil spots at the invasive front of tumor (Fig. S1C).

Given that neutrophil accumulation often correlates with an immunosuppressive TME [28], we utilized functional scores of T cells and macrophages [35, 36] to assess immune activity in each sample type (Fig. 1G). GBC-LI and adjacent livers demonstrated higher M2 score in macrophages and elevated dysfunctional score in T lymphocytes compared to GBC-Lo and GB, indicating a more suppressive immune character for metastatic sites of GBC-LI (Fig. 1G). Furthermore, among 10 macrophage and 11 T lymphocyte clusters (Figs. S1D and S1E), CD4-FOXP3 and Mø-SPP1 showed the highest preference in the tumor samples of GBC-LI (Fig. 1H). CD4-FOXP3 served as the typical regulatory T cells (Tregs) and exhibited the highest dysfunctional score among T lymphocyte clusters (Fig. S1F). Mø-SPP1 presented as a typical protumor M2 Tumor-associated macrophages (TAMs) and preferentially enriched in metastatic sites (GBC-LI), consistent with previous studies [24, 29, 37] (Figs. 1H and S1F). The high proportion of Tregs and M2 TAMs generally suggests an immunosuppressive TME and is associated with poor prognosis in GBC, as demonstrated in previous studies [20].

Neutrophils have been reported to possess immunosuppressive properties in advanced cancer and at metastatic sites [28, 38, 39]. Moreover, they have been found to promote the recruitment of Tregs and M2 TAMs in hepatocellular carcinoma (HCC) [40]. In tissue sections of GBC-LI, we observed co-localization of neutrophil-Treg and neutrophil-M2 populations at the invasive margin of GBC-LI, with neutrophils exhibiting deeper infiltration into the tumor core (Fig. S1G). Using CellPhoneDB [41], we evaluated the interactions between neutrophils, T cells, and macrophages (Fig. S1H). Notably, among the prominent neutrophil-T cell connections enriched in GBC-LI, we identified CCL4L2-VISTA, which has been demonstrated to dampen cytotoxic T-cell responses and foster Treg differentiation [42]. In terms of neutrophil-macrophage interactions, we found that MIF-CD74 was markedly upregulated in GBC-LI, a factor known to facilitate the M2 polarization of TAMs [43]. In all, we discovered the suppressive immune context of GBC-LI, which was characterized by the high infiltration of neutrophils, accompanied with Tregs and M2 TAMs.

### The context-dependent transcriptional states of neutrophils in GBC

**Fig. 2:**
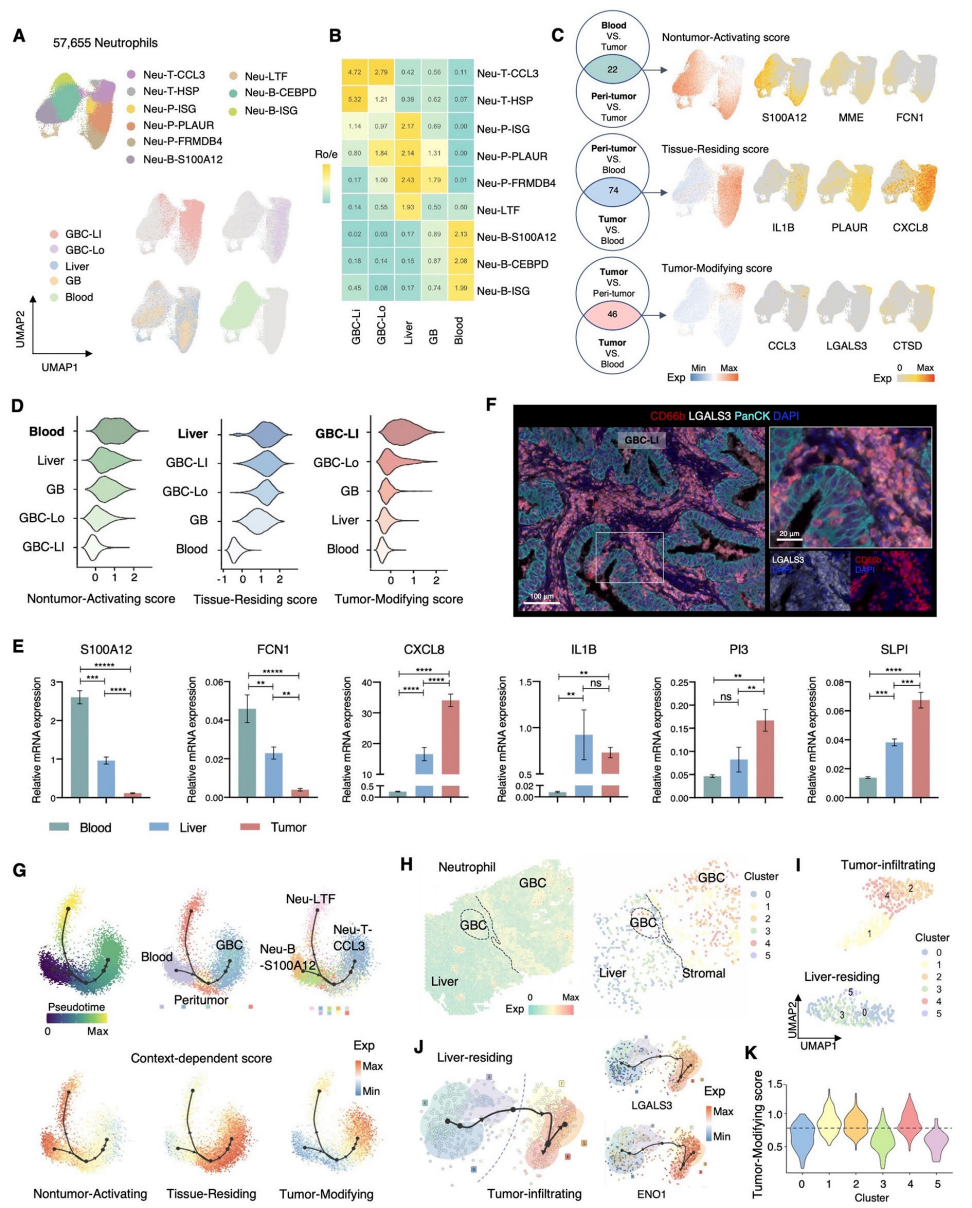
The context-dependent transcriptional states of neutrophils in GBC. (**A**) UMAP of neutrophils from 15 GBC patients, colored by cluster identity (top) and tissue origin (bottom). (**B**) Tissue preference of neutrophil clusters was estimated by the ratio of observed to expected cell numbers (Ro/e) [65]. (**C**) Left, Venn Diagrams showing the number of shared differentially-expressed genes of each sample type for generating the context-dependent scores: Nontumor-Activating, Tissue-Residing, and Tumor-Modifying score; right, UMAP plots showing the expression of overall genes and the representative genes of the context-dependent scores. (**D**) Violin plots displaying the expression of Nontumor-Activating (left), Tissue-Residing (middle), and Tumor-Modifying (right) scores among sample types, ordered by the descending level of expression. (**E**) Relative mRNA expression of representative genes in neutrophils isolated from the blood, peritumoral liver, and tumor samples of GBC-LI patients as measured by qRT-PCR. Statistical analyses were performed using one-way ANOVA with Bonferroni’s multiple comparisons test. (**F**) Representative mIHC images of LGALS3 + neutrophils in the tumor area of GBC-LI. (**G**) Slingshot trajectory plots of all neutrophils showing the pseudotime, tissue origins, cluster identities, and expression of the context-dependent scores. (**H-K**) Analysis of neutrophils on ST-02. Expression of neutrophil signature and the extracted neutrophil spots (**H**). Unsupervised clustering of neutrophil spots (**I**). Slingshot trajectory of neutrophil spots showing the clusters and the expression of Tumor-Modifying genes LGALS3 and ENO1 (**J**). Violin plot showing the expression level of Tumor-Modifying score in each neutrophil cluster (**K**) Data are presented as mean with SD. *P < 0.05, **P < 0.01, ***P < 0.001, ****P < 0.0001 See also Figure S2

While the impact of neutrophils on tumor progression has been demonstrated in many cancer types, their role in GBC remains undiscovered. To get more information of neutrophils, we employed fluorescence-activated cell sorting (FACS) to isolate CD33^mid^ CD66b^+^ neutrophils from the samples of three GBC patients by (two GBC-LI, one GBC-Lo) (Fig. S2A). We adjusted filtering thresholds to accommodate the low transcript counts of neutrophils (averaging 623 genes per cell; other immune cells: 1,569 genes per cell) [44]. Together with neutrophils identified from other samples, clustering 57,665 neutrophils from 39 samples across 15 patients revealed 9 clusters with tissue-specific distribution (Figs. 2A and 2B, and S2B, Table S1).

Neutrophils tended to cluster together according to their tissue origin on the UMAP plots, in contrast to other immune cells such as T cells and macrophages, which did not exhibit distinct clustering patterns that correlated with their tissue of origin (Figs. 2A and 2B, S1D, and S1E). These suggest that the transcriptional states of neutrophils are significantly influenced by their local environment, resulting in a continuous transition within and among tissues. Therefore, we aimed to develop context-dependent scores to assess neutrophil states in GBC. We compared the transcriptional profiles of neutrophils across tumor (GBC-LI and GBC-Lo), peritumor (Liver and GB), and blood samples. By identifying the intersection of differentially-expressed genes (DEGs) from each sample type, we generated three scores (Figs. 2C, S2C, and S2D, Methods, Additional file 1): the Nontumor-Activating score (S100A12, FCN1, MME, etc.), representing the shared signature of neutrophils in blood and peritumoral tissues; the Tissue-Residing score (IL1B, PLAUR, OSM, etc.), reflecting common features among neutrophils in both tumor and peritumoral tissues compared to blood samples; and the Tumor-Modifying score (CCL3, LGALS3, PI3, CTSD, MIF, etc.), derived from the tumor-specific genes in neutrophils, illustrating the unique transcriptional profile of tumor-infiltrated neutrophils.

Among sample types, neutrophils from blood, peritumor liver, and GBC-LI exhibited the highest Nontumor-Activating, Tissue-Residing, and Tumor-Modifying scores, respectively (Figs. 2D, S2E, and S2F). This expression pattern was confirmed by qRT-PCR analysis of neutrophils isolated from corresponding sample types (Fig. 2E). Additionally, we observed the predominance of Tumor-Modifying neutrophils, characterized by LGALS3 expression, in the tumor area on sections of GBC-LI (Fig. 2F). The Slingshot trajectory [6] analysis of neutrophil clusters revealed a predominantly linear trajectory (left to right) from early to late pseudotime, indicating the sequence of neutrophils from blood to peri-tumor and tumor regions (Fig. 2G). This context-dependent acquisition of Tumor-Modifying state in neutrophils was also demonstrated by ST analysis. On the ST feature plots of GBC-LI, neutrophil spots were identified on both the liver and tumor sides, with distinct clustering observed on UMAPs (Figs. 2H and 2I). Again, the trajectory analysis revealed a transition from liver-residing to tumor-infiltrating neutrophils, accompanied by an increase in the Tumor-Modifying score. This indicates a functional transition in neutrophils during their infiltration from the liver to GBC (Figs. 2J and 2K). Moreover, when applying our context-dependent scores to neutrophils from scRNA-seq data of CRC liver metastasis [24] and intrahepatic cholangiocarcinoma [32], the scores effectively described the continuous transcriptional state of neutrophils from nontumor to tumor tissues, indicating a universal pattern of context-dependent states for neutrophils in human cancer (Fig. S3A).

It is noteworthy that within tumor samples, neutrophils from GBC-LI exhibited a higher Tumor-Modifying score compared to those from GBC-Lo, indicating an association between this score and the more advanced stage of GBC (Fig. 2D). This is further supported by RNA-seq data from a previous study, showing that genes from the Tumor-Modifying score displayed higher expression levels in advanced GBC compared to early stages and chronic cholecystitis [45] (Fig. S3B). Additionally, we utilized the pan-cancer data in PRECOG (PREdiction of Clinical Outcomes from Genomic profiles) [46] to estimate the prognostic association for the signature genes in the Nontumor-Activating, Tissue-Residing, and Tumor-Modifying scores. As expected, most genes in the Tumor-Modifying scores negatively correlated with patient survival, while the Tissue-Residing score and the Nontumor-Activating scores generally acted as a neutral indicator (Fig. S3C). These results indicate a universal association between the Tumor-Modifying score of neutrophils and poor prognosis in cancer patients.

### A tumor-modifying state of neutrophils associated with cholesterol metabolism

**Fig. 3:**
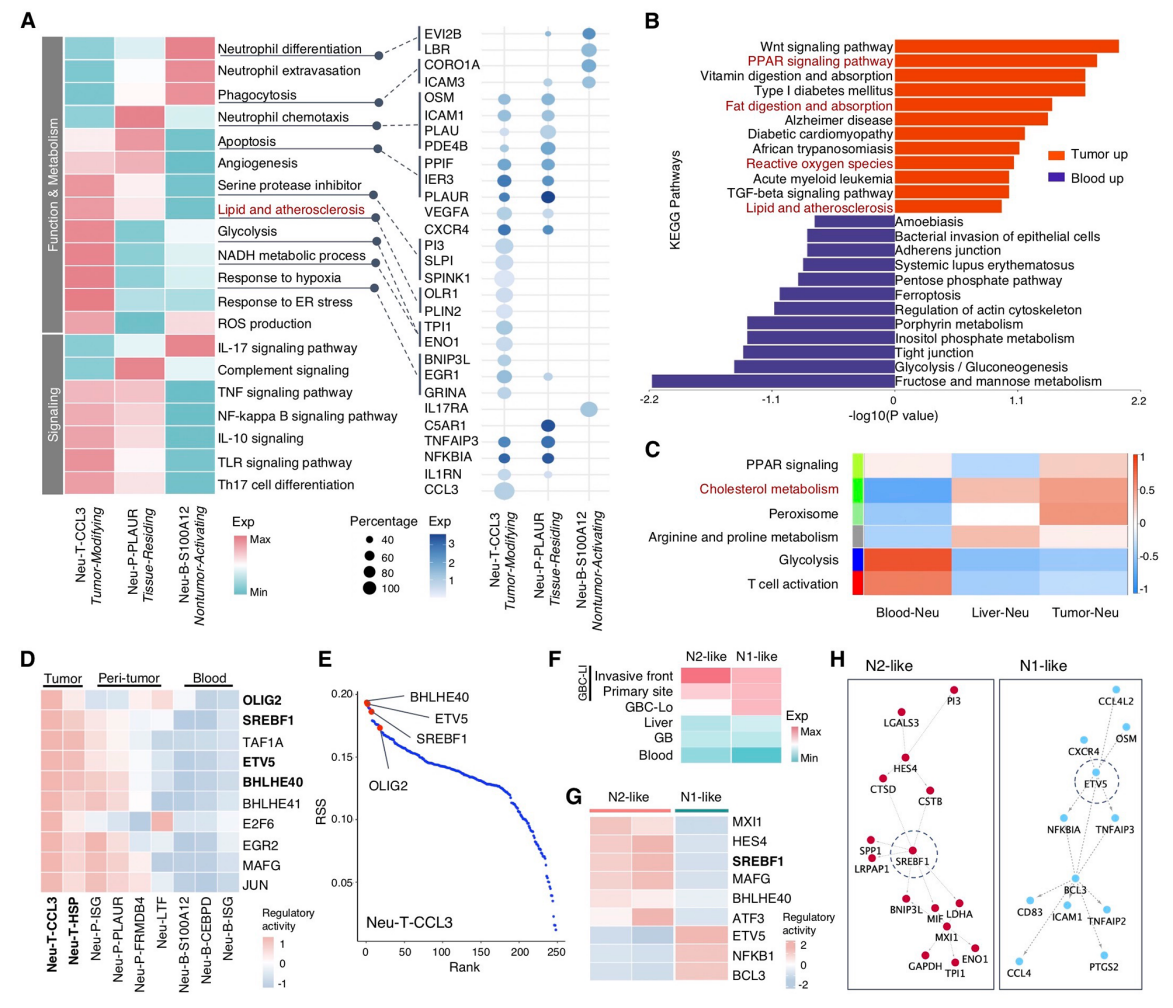
A tumor-modifying state of neutrophils associated with cholesterol metabolism. (**A**) Left, heatmap showing the expression of pathways enriched in the representative neutrophil clusters; right, DotPlot displaying the expression level and percentage of relevant genes. (**B** and **C**) Mass spectrometry-based proteomic data of neutrophils from 3 GBC-LI patients. Top upregulated and downregulated pathways enriched in neutrophils from tumor and blood (**B**). Heatmap of the Module-Trait relationship for neutrophils of blood, liver, and tumor (**C**). (**D** and **E**) SCENIC analysis of neutrophil clusters based on scRNA-seq data. Heatmap displaying the predicted regulatory activity of top 10 regulons for Neu-T-CCL3 (**D**). RSS for Neu-T-CCL3, the top transcriptional factors were annotated (**E**). (**F-H**) Analysis of N1- and N2-like neutrophils within purified Tumor-Modifying neutrophils. Heatmap showing expression of the N1- and N2-like score for neutrophils of different sites (**F**). Heatmap displaying the top regulons for N1- and N2- like neutrophils, colored by the regulatory activity based on SCENIC analysis (**G**). Representative transcriptional factors related to N2-like and N1-like phenotypes and their targeted genes (**H**) See also Figures S3 and S4

For further functional insights into context-dependent states, we compared the profiles of representative neutrophil clusters (Figs. 3A, S3D, and S3E). Blood Neu-B-S100A12, representing a typical Nontumor-Activating state, expressed common surface markers of circulating mature neutrophils (CD10/MME and CD62L/SELL), as well as a range of DAMP (S100A12, S100A4, S100A8, and S100A9) and PAMP (FCN1) molecules essential for neutrophil function in innate immune defense. The liver-enriched Neu-P-PLAUR cluster represents the Tissue-Residing state, characterized by a unique elevation of adhesive and migration genes (C5AR1, ICAM1, and PDE4B), potentially facilitating the high motility of neutrophils in peritumoral tissues as previously reported [47]. While neutrophils originating from nontumor sites shared certain characteristics, those infiltrating tissues exhibited overall distinct features from blood neutrophils. The increase in Tissue-Residing score correlated with the acquisition of aging phenotypes such as CXCR4^hi^ and SELL/CD62L^low^, as well as the upregulation of pro-inflammatory/chemotactic (IL1B, CXCL8, and OSM), angiogenic (VEGFA), and apoptotic (PPIF, IER3, TNFAIP3, and PLAUR) regulators which is associated with the activation of NFKB and TNF signaling pathways. Among the tissue-infiltrated populations, the Neu-T-CCL3 cluster showed preferential enrichment in GBC-LI tumors, representing the Tumor-Modifying state. It is characterized by the expression of monokines (CCL3, CCL4, CCL3L1 and CCL4L2), inhibitory factors (MIF, IL1RN), enzymatic factors (PI3, SLPI, and CTSD), glycolytic enzymes (ENO1, TPI1), and genes involved in the hypoxic response (GRINA, EGR1, and BNIP3L), endoplasmic reticulum (ER) stress, reactive oxygen species (ROS) production, and lipid and atherosclerosis (OLR1, PLIN2), reflecting the unique metabolic adaptation in response to the TME (Figs. 3A, S3D, and S3E). Additionally, we noted that most macrophages exhibit a high expression of the Tumor-Modifying score, indicating a potential shift towards a macrophage-like phenotype in tumor-infiltrated neutrophils (Fig. S3F).

Due to the inherent low mRNA abundance in neutrophils, we complemented the neutrophil sequencing with mass spectrometry-based proteomics, identifying a total of 17,018 peptides and 2,230 proteins in neutrophils from tumor, liver, and blood samples of 3 GBC-LI patients (Methods). Differentially expressed proteins in tumor-neutrophils were enriched in pathways related to lipid and cholesterol metabolism, including the lipid and atherosclerosis pathway (Fig. 3B). Module-trait relationships assigned “Cholesterol metabolism” and “Peroxisome” to tumor-neutrophils as unique traits (Fig. 3C). Overall, our findings at both transcriptome and proteome levels suggest heightened expression of genes related to cholesterol metabolism in neutrophils within the tumor tissue of GBC-LI.

Then, we conducted SCENIC analysis to explore the transcriptional regulation driving the acquisition of the cholesterol-related Tumor-Modifying state in neutrophils [8] which revealed activation of BHLH family transcription factors (BHLHE40, ETV5, OLIG2, and SREBF1) within the Neu-T-CCL3 cluster (Figs. 3D, 3E, S4A, and S4B). Additionally, we identified a subtype of unfavorable N2-like neutrophils [48] within the Neu-T-CCL3 cluster, which were preferentially enriched in the invasive front of GBC-LI, and characterized by signature associated with the metabolic adaptation to tumor context (Figs. S4C-F). SCENIC analysis further highlighted the activation of SREBF1 in these N2-like neutrophils, linking lipid and cholesterol metabolism with the terminal state of GBC-infiltrated neutrophils (Figs. 3G and 3H).

### GBC-neutrophil contact induces oxLDL uptake in neutrophils

**Fig. 4:**
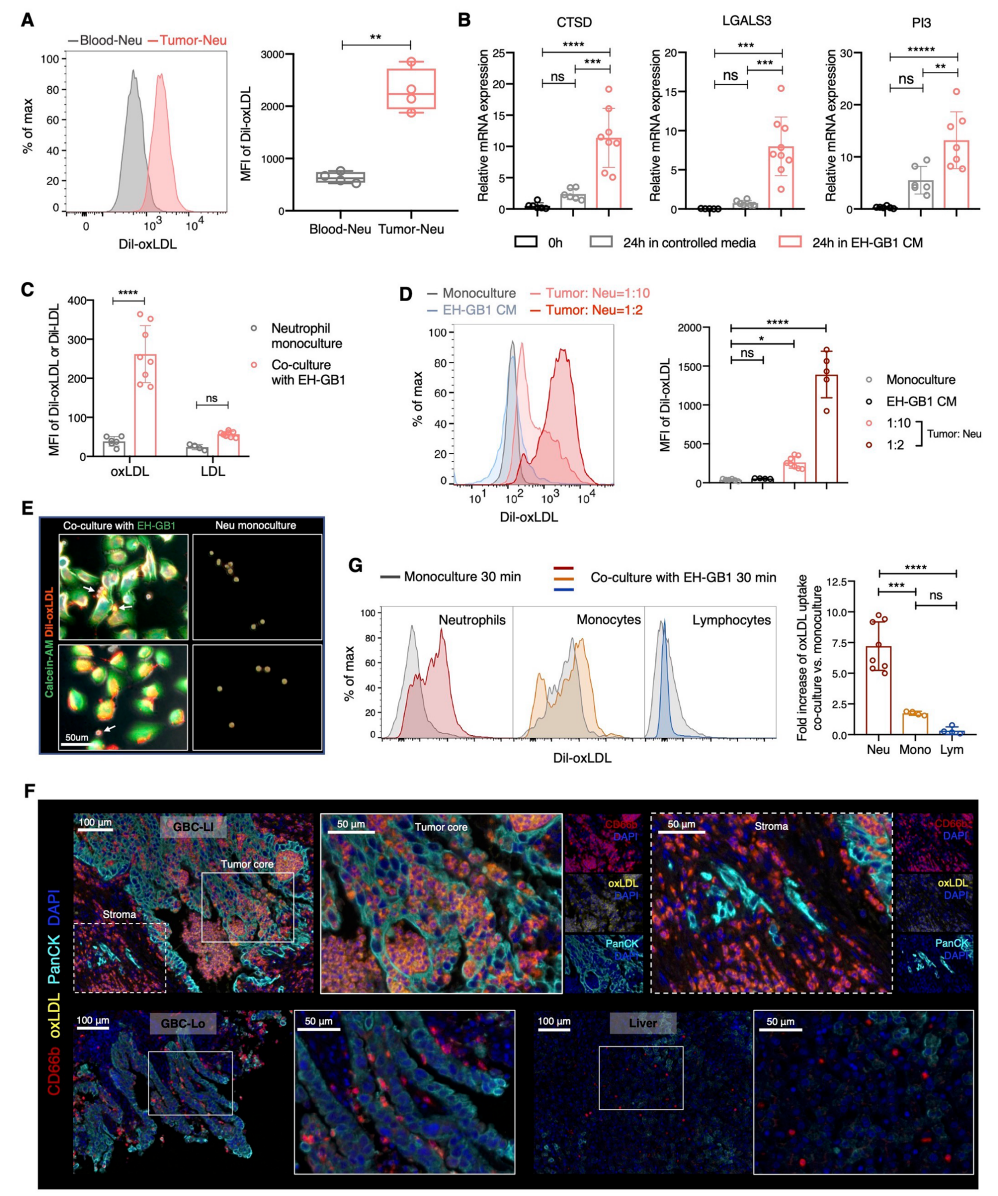
GBC-neutrophil contact induces oxLDL uptake in neutrophils. (**A**) Representative histogram and quantification showing the oxLDL uptake by neutrophils from tumor and blood of GBC patients (*n* = 4) as measured by flow cytometry. Comparison was performed using two-tailed unpaired t-test. (**B**) Relative mRNA expression of CTSD, LGALS3 and PI3 in blood neutrophils before and after cultured in controlled media or EH-GB1 conditioned media (CM) for 24 h as measured by qRT-PCR. Statistical analyses were performed using one-way ANOVA with Tukey’s multiple comparisons test. (**C**) Quantification of oxLDL or LDL uptake by neutrophils after monoculture or co-cultured with EH-GB1 for 30 min as measured by flow cytometry. Statistical analyses were performed using two-way ANOVA with Sidak’s multiple comparisons test. (**D**) Representative histogram and quantification showing the oxLDL uptake of neutrophils after monoculture, cultured in EH-GB1 CM, or co-cultured with EH-GB1 at different Tumor: Neu ratio for 30 min as measured by flow cytometry. One-way ANOVA with Tukey’s multiple comparisons test. (**E**) Representative microscopic images of oxLDL-absorbing neutrophils (white arrow). EH-GB1 cells were pre-labeled with calcein-AM. (**F**) Representative mIHC staining of oxLDL-absorbing neutrophils on tissue sections of GBC patients. (**G**) Representative histogram and quantification showing the oxLDL uptake of neutrophils, monocytes, and lymphocytes after monoculture or co-cultured with EH-GB1 for 30 min as measured by flow cytometry. One-way ANOVA with Bonferroni’s multiple comparisons test Data are presented as mean with SD. *P < 0.05, **P < 0.01, ***P < 0.001, ****P < 0.0001 See also Figure S5

GBC-infiltrated neutrophils exhibit a distinct signature associated with cholesterol metabolism, particularly in pathways related to “Lipid and Atherosclerosis,” where oxLDL plays a pivotal role in disease progression. As observed in atherosclerosis, oxLDL accumulation within macrophages triggers foam cell formation and activates inflammatory genes [49]. Given our identification of a macrophage-like signature in GBC-infiltrated neutrophils (Fig. S3F), it raises the possibility of these neutrophils possessing a similar capacity for oxLDL uptake. Therefore, we hypothesize that oxLDL may contribute to the induction of the cholesterol-related GBC-infiltrated neutrophils. As anticipated, we observed a higher oxLDL uptake in neutrophils isolated from GBC tissues compared to blood neutrophils through flow cytometry analysis (Fig. 4A). To investigate how neutrophils acquire the capacity of absorbing oxLDL, we at first cultured blood neutrophils with the conditioned media (CM) derived from the GBC cell line EH-GB1 for 24 h. However, despite the upregulation of several Tumor-Modifying genes, increased ROS production, and prolonged survival, the oxLDL uptake of neutrophils did not show a substantial increase (Figs. 4B and S5A-C). Surprisingly, direct co-culture with EH-GB1 resulted in a rapid and substantial increase in oxLDL uptake by neutrophils, surpassing fivefold within 30 min (Fig. 4C). On the other hand, while we also observed an increase in LDL uptake by neutrophils, it was not as pronounced as the increase in oxLDL uptake (Figs. 4C and S5D). Furthermore, we observed a consistent correlation between the extent of neutrophil oxLDL uptake enhancement and the Tumor: Neutrophil ratio (Fig. 4D). When the Tumor: Neutrophil ratio was relatively low at 1:10, neutrophil oxLDL uptake increased by 7-fold. With the ratio elevated to 1:2, the increase in oxLDL uptake was further amplified to 30-fold. These findings suggest the critical role of cell-cell contact, rather than soluble factors, in the induction of oxLDL-absorbing neutrophils by GBC. Microscopic examination revealed that the majority of oxLDL-absorbing neutrophils labeled with Dil-oxLDL were attached to GBC cells, whereas unattached neutrophils or those in monoculture showed minimal labeling with Dil-oxLDL (Fig. 4E). Further analysis via mIHC staining demonstrated the presence of oxLDL-absorbing neutrophils in GBC-LI tumor tissues (Fig. 4F). Neutrophils tended to colocalize with oxLDL when deeply infiltrated into the tumor core, whereas neutrophils in other tissue areas, such as the stroma and liver, exhibited minimal staining with oxLDL. These results underscore the dependence of oxLDL-absorbing neutrophil induction on GBC-neutrophil contact. Additionally, we sought to investigate whether other immune cells exhibited a similar response as neutrophils. Lymphoid cells showed minimal oxLDL uptake both before and after co-culture, suggesting a lack of significant influence from the GBC cells. Similarly, although monocytes displayed high oxLDL uptake when in monoculture, co-culture with EH-GB1 only mildly promoted their oxLDL absorption (Figs. 4G and S5E-G). These results further confirmed the uniqueness of neutrophils response to direct cell-cell contact with GBC cells.

### OLR1 mediates the oxLDL uptake of pro-tumor neutrophils

**Fig. 5:**
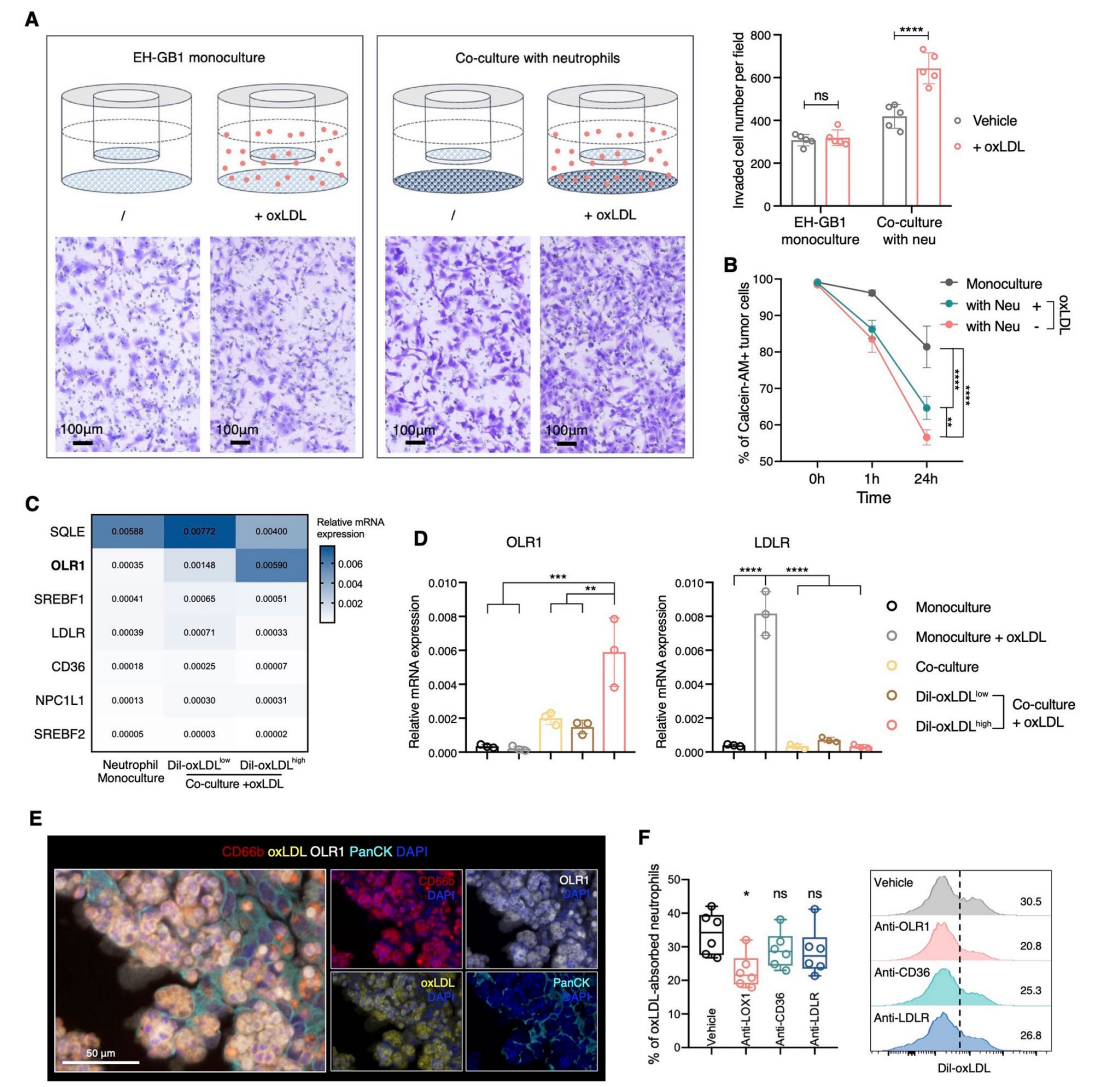
OLR1 mediates the oxLDL uptake of pro-tumor neutrophils. (**A**) Transwell invasion assay, showing the representative images and quantification of the invaded GBC cells. Cells were counted in each 20x field-of-view image. Two-way ANOVA with Sidak’s multiple comparisons test. (**B**) Percentage of viable GBC cells labeled with calcein-AM after monoculture or co-cultured with neutrophils with or without oxLDL supplement for 1 h and 24 h as measured by flow cytometry. Two-way ANOVA with Sidak’s multiple comparisons test. (**C**) Heatmap displaying the relative mRNA expression of cholesterol-related genes in monoculture neutrophils, Dil-oxLDL^low^, and Dil-oxLDL^high^ neutrophils as measured by qRT-PCR. (**D**) Quantification of relative mRNA expression of OLR1 and LDLR in neutrophils when monoculture or co-culture with or without oxLDL addition. Statistical analyses were performed using one-way ANOVA with Tukey’s multiple comparisons test. (**E**) Representative mIHC staining of OLR1 + oxLDL-absorbing neutrophils on sections of GBC-LI. (**F**) Representative histogram and quantification showing the percentage of oxLDL-absorbing neutrophil when co-cultured with EH-GB1 cells with treatment of different antibodies of common receptors for cholesterol. One-way ANOVA with Tukey’s multiple comparisons test Data are presented as mean with SD. *P < 0.05, **P < 0.01, ***P < 0.001, ****P < 0.0001 See also Figure S6

As the majority of oxLDL-absorbing neutrophils were found in GBC-LI, we hypothesized that they might influence tumor invasion. In the transwell invasion assay, we simulated a scenario to observe how the interaction between GBC cells, neutrophils, and oxLDL at metastatic sites affects subsequent invasion (Fig. 5A). Addition of neutrophils to the bottom chamber (representing the metastatic site) indeed led to an increase in tumor invasion, while the addition of oxLDL alone did not. However, in the presence of neutrophils, the addition of oxLDL had a significantly greater effect on promoting GBC cell invasion, suggesting the pivotal role of oxLDL-absorbing neutrophils in this process (Fig. 5A). This pro-metastatic effect may be attributed to soluble factors, as evidenced by the conditioned media generated from the GBC-neutrophil co-culture system supplemented with oxLDL, which also induced significant tumor invasion and resulted in an even larger disparity (Fig. S6A). Lipid peroxidation and ferroptosis, known to contribute to the pro-metastatic effects of neutrophils [50], were also evident in oxLDL-absorbing neutrophils, as indicated by a significant increase in lipid ROS levels (Fig. S6B). Then we also investigate the cancer cytotoxicity derived by the oxLDL-absorbing neutrophils. After 24 h of co-culture, we observed that neutrophils caused less apoptosis in GBC cells in the presence of oxLDL, as shown by Annexin V staining (Fig. S6C). Additionally, co-culture with neutrophils resulted in a significant decrease in GBC cell viability, as evidenced by the reduction in calcein-AM levels, possibly attributable to neutrophil-mediated trogoptosis [51]. The addition of oxLDL partially restored GBC cell viability, indicating reduced cytotoxicity of oxLDL-absorbing neutrophils (Fig. 5B). Collectively, these results show that oxLDL-absorbing neutrophils acquired a higher capacity to promote cancer invasion while displaying lower cancer cytotoxicity.

To gain deeper insights into the molecular characteristics of oxLDL-absorbing neutrophils, we purified Dil-oxLDL^high^ and Dil-oxLDL^low^ neutrophil subpopulations by FACS after co-culture with GBC cells. Subsequently, we assessed the mRNA expression levels of several genes related to cholesterol transport and biosynthesis using qRT-PCR (Fig. 5C). We found that OLR1 was the most significantly upregulated gene in oxLDL-absorbing neutrophils (Fig. 5D). Instead, other cholesterol-related genes like SREBF1, SQLE, LDLR, and NPC1L1 exhibited substantial upregulation in monoculture neutrophils incubated with oxLDL, but with a particularly low oxLDL uptake capacity (Figs. 5D and S6D). This suggests that the expression of these genes relies on extracellular oxLDL stimulation. We further used flow cytometry to measure the expression of these cholesterol-related markers, confirming the elevated expression of OLR1 in oxLDL-absorbing neutrophils (Fig. S6E). MIHC analysis of GBC-LI sections also revealed OLR1 staining on oxLDL + neutrophils within the tumor area (Fig. 5E). Blocking OLR1 on neutrophils with anti-OLR1 antibodies led to a substantial decrease in oxLDL uptake, with other oxLDL receptors showing limited efficacy in inhibition (Fig. 5F). Overall, these findings indicate that GBC-neutrophil contact induces OLR1-mediated oxLDL uptake in neutrophils, leading to a pro-tumor phenotype.

### KRT17^+^ GBC cells cultivate the oxLDL-absorbing neutrophils

**Fig. 6:**
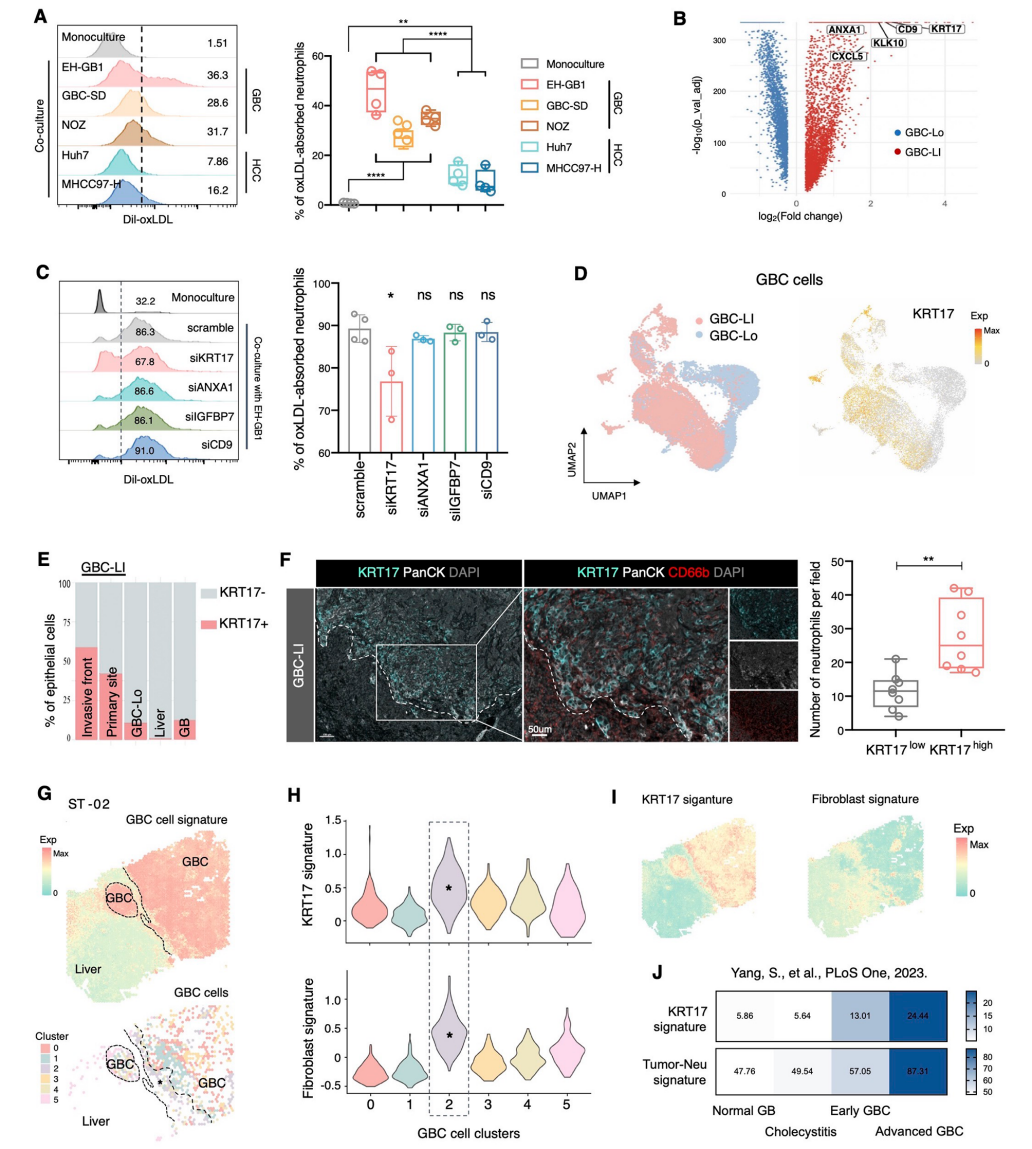
KRT17^+^ GBC cells cultivate the oxLDL-absorbing neutrophils. (**A**) Representative histogram and quantification showing the percentage of oxLDL-absorbing neutrophils induced by different GBC and HCC cell lines. One-way ANOVA with Tukey’s multiple comparisons test. (**B**) Volcano plot showing the differentially expressed genes between tumor cells from GBC-LI and GBC-Lo as measured by scRNA-seq analysis. The fold change of gene expression (GBC-LI versus GBC-Lo) and adjusted *P* values (Benjamini-Hochberg method) are shown. (**C**) Representative histograms and quantifications showing the percentage of oxLDL-absorbing neutrophils induced by monoculture or co-culture with EH-GB1 of siRNA knockdown of KRT17, ANXA1, IGFBP7, and CD9 respectively. Statistical analyses were performed using one-way ANOVA with Bonferroni’s multiple comparisons test. (**D**) UMAP of GBC cells colored by sample origin (left) and showing the expression of KRT17 (right). (**E**) Percentage of KRT17^+^ and KRT17^-^ cells in all epithelial cells from different types of samples. (**F**) Left, representative mIHC images of KRT17^+^ GBC cells and CD66b^+^ neutrophils on section of GBC-LI (*n* = 5). The dash line separated the tumor and non-tumor area. Right, quantification of the neutrophil numbers in each KRT17^high^ and KRT17^low^ region (20x field-of-view, stratified by the 50% staining of KRT17 in PanCK^+^ GBC cells). Comparison was performed using two-tailed unpaired t-test. (**G-I**) Analysis of GBC cells on ST-02. Expression of GBC cell signature (top) and the extracted GBC cell spots (colored by cluster identity) (bottom) (**G**). Violin plots showing the expression of KRT17 signature (top) and fibroblast signature (bottom) in each GBC cell cluster, the annotated cluster is the KRT17-Fibroblast high cluster (**H**). Expression of KRT17 and fibroblast signature (**I**). (**J**) Histograms showing the average expression of KRT17 signature and Tumor-Modifying score of neutrophils in different tissue samples (normal GB, *n* = 3, chronic cholecystitis, *n* = 4; early GBC, *n* = 5; advanced GBC, *n* = 5) Data are presented as mean with SD. *P < 0.05, **P < 0.01, ***P < 0.001, ****P < 0.0001 See also Figure S7 and Figure S8

ScRNA-seq data and mIHC staining confirmed the prevalence of oxLDL^+^ neutrophils in GBC-LI. Co-culture experiments showed that GBC cells (EH-GB1, GBC-SD, and NOZ) induced higher oxLDL uptake and OLR1 expression in neutrophils compared to HCC cells (Huh7 and MHCC97-H), especially EH-GB1 from metastatic gallbladder cancer (Figs. 6A and S7A). These results suggest distinctive traits of GBC-LI cells in reprogramming neutrophils.

First, we began by investigating the mutational features of GBC-LI. Whole-exome sequencing data revealed typical genomic mutation events in GBC [16], with over 70% of tumors harboring mutated TP53 (Fig. S7B). No differential mutational patterns were observed between GBC-LI and GBC-Lo patients. Then, we turned our focus to scRNA-seq data. Utilizing inferCNV [4], we successfully discriminated 20,284 malignant epithelial cells (GBC cells) from normal cells within 16 tumor samples obtained from 12 GBC patients (Fig. S7C, Methods). Analysis of the DEGs (Fig. 6B) revealed that GBC cells from GBC-LI were enriched with genes associated with motility and metastatic potential, including keratins (KRT17, KRT6), mesenchymal markers (EDN1, MYL9), and cell adhesion and wound healing markers (IGFBP7, CD9, FLINA, FSTL1, and FLOR1). Additionally, we detected the expression of ANXA1, SAA1, CXCL5, and S100-family members, known to participate in neutrophil chemotaxis and degranulation. The slingshot trajectory of GBC cells also displayed an increasing gene expression associated with cell adhesion and neutrophil activation from GBC-Lo to GBC-LI (Fig. S7D). These results suggested the invasiveness and the potential role in affecting neutrophil behaviors of GBC-LI cells.

To identify the critical gene responsible for inducing neutrophil oxLDL uptake in GBC cells, we conducted siRNA knockdown on several top DEGs of GBC-LI cells (KRT17, ANXA1, IGFBP7, CD9) in EH-GB1 before co-culturing with neutrophils (Figs S7E and S7F). Remarkably, only knockdown of KRT17 (siKRT17) in EH-GB1 significantly suppressed the upregulation of neutrophil oxLDL uptake and induced fewer OLR1^+^ neutrophils during co-culture (Fig. 6C, S7F and S7G). Additionally, it’s worth noting that the expression of KRT17 in EH-GB1 cells was notably higher than that in GBC-SD and NOZ cells, which aligns with their varying abilities to induce neutrophil oxLDL uptake (Figs. 7A and S7H). Based on our scRNA-seq data, KRT17 exhibited significantly higher expression levels in GBC-LI compared to GBC-Lo (Figs. 6D and S7I), with the highest proportion in the invasive front of GBC-LI among all sample types (Fig. 6E). This spatial distribution pattern was confirmed on GBC-LI sections by mIHC, and further revealing the preference of neutrophils in KRT17^hi^ tumor area (Fig. 6F). These findings suggest the pivotal role of KRT17 in mediating the induction of neutrophil oxLDL uptake by GBC cells.

Consequently, we constructed a KRT17 signature consisting of 19 genes that included DEGs from GBC-LI and genes positively correlated with KRT17 (Additional file 1). Application of this signature on ST plots demonstrated that epithelial-enriched spots with high KRT17 signature expression also exhibited elevated levels of fibroblast-related genes (Figs. 6G-I). MIHC of GBC-LI sections further revealed the CAF-enriched KRT17^+^ tumor region with a high neutrophil infiltration (Fig. S6J). Taken together, these results depicted a possible neutrophil niche consisting of KRT17^+^ GBC cells and CAFs, which help cultivate the oxLDL-absorbing neutrophils and promote GBC progression.

Finally, as we investigated the prognostic significance of KRT17, we at first recognized that the high KRT17 expression predicted a worse survival in bile duct cancer (TCGA_CHOL) and linked with the perineural invasion (Figs. S8A and S8B). Based on RNA-seq data from various stages of GBC and from gallbladders affected by chronic cholecystitis [45], both the KRT17 signature and the Tumor-Modifying score of neutrophils exhibited similar expression patterns across different sample types, with distinct elevation observed specifically in advanced GBC (Figs. 6J and S8C). Additionally, we also observed a positive correlation between the expression of KRT17 signature with the neutrophil signature across various cancer types (Fig. S8D). Notably, high KRT17 signature expression predicted a worse survival in KRT17^hi^ cancer type like pancreatic adenocarcinoma (TCGA_PAAD). Conversely, in cancer types where the expression of the KRT17 signature was relatively low, such as liver cancer (TCGA_LIHC), it did not serve as an indicator of survival (Figs. S8E and S8F).

## Discussion

A growing body of evidence has shown the critical role of neutrophils in the TME, especially in their accumulation in (pre) metastatic niches and active engagement in the process of tumor progression [28]. Previously considered short-lived and homogenous, recent research has revealed that neutrophils can persist in tissue, especially within tumor environments, for extended periods, allowing them to respond to environmental cues and undergo adaptive epigenetic and transcriptional reprogramming [52]. With increasing recognition of the functional diversity and plasticity of neutrophils in cancer, there is a growing need to characterize the cellular states of neutrophils within specific tissue and tumor contexts [38, 44]. ScRNA studies have highlighted the high heterogeneity of neutrophils across various tissues and diseases [44, 53–55], yet the dynamic spectrum of neutrophil phenotypes in gallbladder cancer (GBC) remains insufficiently defined.

While advanced GBC has traditionally been associated with enriched populations of Tregs and M2-macrophages, our study integrates neutrophils into this suppressive immune landscape. Through scRNA-seq analysis, we elucidate context-dependent transcriptional states of neutrophils in GBC patients across multiple stages, revealing a distinct terminal tumor-infiltrated neutrophil state associated with lipid and cholesterol metabolism. In vitro experiments employing GBC-neutrophil co-culture and oxLDL stimulation faithfully mimic this cholesterol-related neutrophil phenotype, and further confirmed the pro-tumoral capacity of these oxLDL-absorbing neutrophils. The uptake of neutrophil oxLDL is facilitated by OLR1 activation triggered by direct cell-to-cell contact between GBC cells and neutrophils. Additionally, we identify KRT17 ^+^ GBC cells as crucial components for fostering oxLDL-absorbing neutrophils. Moreover, we establish an association between the Tumor-Modifying score of neutrophils, KRT17 signature of tumor cells, and advanced GBC stage, underscoring their potential as unfavorable prognostic indicators across various cancer types.

ScRNA-seq analysis reveals a distinct tissue-specific distribution of neutrophils among all cell types, with annotation of most clusters lacking clear biological significance. Notably, clusters like Neu-B-ISG and Neu-P-ISG, sharing the interferon-stimulated gene (ISG) signature, are classified into separate clusters based on tissue origin, indicating the context-dependent nature of neutrophil transcriptional states. Therefore, we generate context-dependent scores describing the common biological features of neutrophil clusters from the same sample type, revealing a clear transition of neutrophils from blood to peritumor tissue (gallbladder and liver), and finally to GBC tumor tissue. We further found that these context-dependent scores also effectively described the neutrophil reprogramming from blood to tissue and from non-tumor to tumor environments across multiple cancer types, indicating a consistent pattern of neutrophil behavior in response to specific environmental signals.

Annotation of Neu-T-CCL3 suggests its similarity with previously reported terminal neutrophil clusters (e.g., CCL4 ^+^ TANs [55], TAN-1 [54], and T3 [56]) undergoing reprogramming in response to hypoxic and glycolytic TME. Uniquely, we identified a subset of neutrophils within the Neu-T-CCL3 cluster that exhibits a cholesterol-related phenotype, induced in vitro by direct contact between GBC cells and neutrophils in the presence of oxLDL. Moreover, we validate the restricted presence of oxLDL-absorbing neutrophils within the tumor core of GBC, highlighting intratumoral neutrophil heterogeneity recently demonstrated [56].

Intratumoral immune cells can metabolically adapt to the lipid-enriched TME by enhancing lipid uptake or storage, which, in turn, is associated with a dysfunctional or pro-tumoral state [57]. The oxLDL-absorbing neutrophils exhibit properties facilitating invasion while displaying low cancer cytotoxicity. The pro-metastatic effect is attributed to the soluble factors generated by the GBC-neutrophil coculture system with oxLDL addition, as evidenced by the conditioned media’s ability to induce heightened invasion. Furthermore, oxLDL-absorbing neutrophils notably promote invasion in remote GBC cells rather than those they directly contact. This illustrates a scenario where neutrophils are initially recruited to metastatic sites in the liver, attach to GBC cells, and begin absorbing oxLDL, thereby generating pro-metastatic factors that facilitate subsequent invasion of GBC cells. Multiple mechanisms may be involved in this process, such as oxLDL uptake inducing NETosis or ferroptosis of neutrophils [50, 58]. Additionally, it is plausible that neutrophils in metastatic sites accumulate oxLDL and transport their lipids directly to metastatic tumor cells to meet their energy demands [59, 60]. While the specific mechanisms and substances driving this pro-metastatic effect were not explored in this study due to difficulties in defining cell origin, considering oxLDL’s detrimental role in various diseases and its immunosuppressive effects [61], clearing oxLDL may serve as a therapeutic strategy to prevent the induction of these harmful neutrophils.

Although previous research has demonstrated high expression of LOX-1 (OLR1) on neutrophils within tumors and its association with immunosuppressive and pro-tumoral effects [62], no prior findings have established a connection between this ER stress-induced OLR1 and the oxLDL uptake by neutrophils. In our study, we found that the uptake of oxLDL by neutrophils was primarily mediated through OLR1 on neutrophils. Although OLR1 may be the main mechanism for neutrophil oxLDL uptake, as it outperforms well-studied cholesterol-related molecules like CD36 and LDLR, its inhibition was still unable to entirely block this process. Given the significant alterations in neutrophil properties induced by GBC-neutrophil contact, we speculate that neutrophil oxLDL absorption may involve multiple molecular and cellular changes extending beyond OLR1 activation. One potential explanation could be the transition of tumor-associated neutrophils to a macrophage-like phenotype, as suggested by our single-cell RNA sequencing data. Nevertheless, OLR1 expression remains a distinctive marker for oxLDL-absorbing neutrophils enriched in GBC-LI.

High levels of cholesterol in the blood have been linked to an increased risk of several types of cancer, including gallbladder cancer [63]. In the case of GBC, cholesterol plays a crucial role in cancer initiation and progression. Elevated cholesterol levels in bile can lead to the formation of crystals and stones, causing damage to the gallbladder lining and resulting in chronic inflammation and subsequent cancerous growth [61]. While both macrophages and CD8 ^+^ T cells have demonstrated the ability to absorb oxLDL and contribute to disease progression [64], neutrophils are the exclusive immune cell type that dramatically activates oxLDL absorption upon direct contact with tumor cells. Moreover, this contact-dependent activation of oxLDL uptake appears to be specific to KRT17^hi^ GBC cells, rather than KRT17^low^ HCC cells. KRT17 might contribute to the induction of oxLDL-absorbing neutrophils, and it is preferentially expressed in advanced GBC and correlates with poorer survival in bile duct cancer and pancreatic cancer. However, KRT17 is not indicative of survival in HCC, likely due to its low expression levels, which may not be sufficient to induce the pro-tumoral oxLDL-absorbing neutrophils. The process of the GBC-neutrophil contact and subsequent oxLDL uptake may involve much more sophisticated mechanism beyond tumor-expressed KRT17 and neutrophil OLR1 activation, which require future exploration. Previous studies have mainly focused on paracrine influences affecting neutrophils, such as the impact of mesenchymal-derived PGE2 in augmenting neutrophil lipid accumulation [59]. In contrast, our study reveal that direct cell-cell contact between GBC cells and neutrophils emerge as a markedly more potent initiator for inducing OLR1-mediated oxLDL uptake by neutrophils. Consequently, our findings suggest the possibility of an expanded repertoire of neutrophil activation following GBC-neutrophil contact, transcending the oxLDL uptake. This heightened plasticity of neutrophils presents an exciting opportunity to exploit their responsiveness to environmental cues and opens new avenues for designing innovative immunotherapeutic strategies.

## Conclusion

We characterized the immune landscape associated with liver invasion in GBC through the integration of scRNA-seq, ST, proteomics, mIHC. Our study comprehensively delineated the context-dependent transcriptional states of neutrophils. We identified a novel state of tumor-infiltrated neutrophils distinguished by OLR1-mediated oxLDL uptake. Notably, the activation of oxLDL uptake was significantly pronounced only upon neutrophil interaction with KRT17 ^+^ GBC cells, which are enriched in GBC liver invasion. Importantly, these oxLDL-absorbing neutrophils demonstrated the ability to enhance the invasion of GBC. These findings offer a novel perspective on targeting tumor-infiltrated neutrophils for early identification and intervention of invasive behaviors in cancer types like GBC.

## Electronic supplementary material

Below is the link to the electronic supplementary material.

Supplementary Material 1

## Data Availability

All data relevant to this study are available from the corresponding author on reasonable request. Sc-RNA, ST, and WES data are available at the GSA (https://ngdc.cncb.ac.cn/gsa-human/): HRA005325

## Abbreviations

CM: Conditioned Media
CRC: Colorectal cancer
DEGs: Differentially expressed genes
GBC-LI: GBC liver invasion
GBC-Lo: Localized GBC
GBC: Gallbladder cancer
HCC: Hepatocellular carcinoma
LIHC: Liver hepatocellular carcinoma
mIHC: Multiplexed immunohistochemistry
OLR1: Oxidized low-density lipoprotein receptor 1
OxLDL: Oxidized low-density lipoprotein
PAAD: Pancreatic adenocarcinoma
qRT-PCR: Quantitative real-time PCR
ScRNA-seq: Single-cell RNA sequencing
ST: Spatial transcriptomics
TCGA: The Cancer Genome Atlas
TME: Tumor microenvironment
