# Correction: Multi-model analysis of gallbladder cancer reveals the role of OxLDL-absorbing neutrophils in promoting liver invasion

**DOI:** 10.1186/s40164-025-00722-8

**Published:** 2025-11-05

**Authors:** Dongning Rao, Jiaxin Li, Mao Zhang, Siyuan Huang, Lu Meng, Guohe Song, Jiaqiang Ma, Yingcheng Wu, Yifei Cheng, Shuyi Ji, Gaohua Wu, Lv Chen, Yuming Liu, Yang Shi, Jian Zhou, Fan Jia, Xiaoming Zhang, Ruibin Xi, Qiang Gao

**Affiliations:** 1 Department of Liver Surgery and Transplantation, Key Laboratory of Carcinogenesis and Cancer Invasion (Ministry of Education), Liver Cancer Institute, Zhongshan Hospital, Fudan University, Shanghai, 200032 China; 2 Peking-Tsinghua Center for Life Sciences, Academy for Advanced Interdisciplinary Studies, Peking University, Beijing, 100871 China; 3 Academy for Advanced Interdisciplinary Studies, Peking University, Beijing, 100871 China; 4 Shanghai Key Laboratory of Infectious Diseases and Biosafety Emergency Response, State Key Laboratory of Genetic Engineering, Institute of Infection and Health, Huashan Hospital, National Medical Center for Infectious Diseases, Fudan University, Shanghai, China; 5 Shanghai Institute of Immunity and Infection, Chinese Academy of Sciences, Shanghai, 200031 China; 6 BeiGene (Beijing) Co., Ltd, Beijing, China; 7 School of Mathematical Sciences, Center for Statistical Science, Peking University, Beijing, China; 8 Key Laboratory of Medical Epigenetics and Metabolism, Institutes of Biomedical Sciences, Fudan University, Shanghai, China; 9 State Key Laboratory of Genetic Engineering, Fudan University, Shanghai, China

**Correction: Experimental Hematology & Oncology (2024) 13:58** 10.1186/s40164-024-00521-7

The data availability statement in the article [1], the reference number and access link for shared data was incorrect. The revised statement with correct reference number and access link is given below.

**Data availability**

All data relevant to this study are available from the corresponding author on reasonable request. Sc-RNA, ST, and WES data are available at the GSA (https://ngdc.cncb.ac.cn/gsa-human/): HRA005325.

The original article has been corrected.
