# Supplementary material for: Multi-model analysis of gallbladder cancer reveals the role of OxLDL-absorbing neutrophils in promoting liver invasion
Source: Exp Hematol Oncol. 2024 May 31;13:58. doi: 10.1186/s40164-024-00521-7 (PMC11140996; doi:10.1186/s40164-024-00521-7)
Supplement: Supplementary file 1 — Supplementary Material 1 [file 40164_2024_521_MOESM1_ESM.docx]

**Supplement Material**

**Supplementary Figures**

**Figure S1.** Characteristics of major immune components in GBC TME, Related to Figure 1.

**Figure S2.** Neutrophil diversity as indicated by the context-dependent scores, Related to Figure 2.

**Figure S3.** Prognostic and Biological significance of the context-dependent scores, Related to Figure 3.

**Figure S4.** Transcriptional factors and the heterogeneity of tumor-neutrophils, Related to Figure 3.

**Figure S5.** Cholesterol uptake of immune cells in response to GBC cells, Related to Figure 4.

**Figure S6.** Alteration of neutrophils in response to oxLDL, Related to Figure 5.

**Figure S7.** Characteristics of KRT17^+^ GBC cells, Related to Figure 6.

**Figure S8.** Prognostic significance of KRT17 signature, Related to Figure 6.

**Supplemental Tables**

**Supplemental Table 1.** Clinical characteristics of GBC patients from the scRNA-seq cohort.

**Supplemental Table 2.** Summary of analytical methods.

**Additional file 1.** Gene lists of immune signatures, context-dependent scores, and the KRT17 signature.

**Additional file 2**. R code.

**
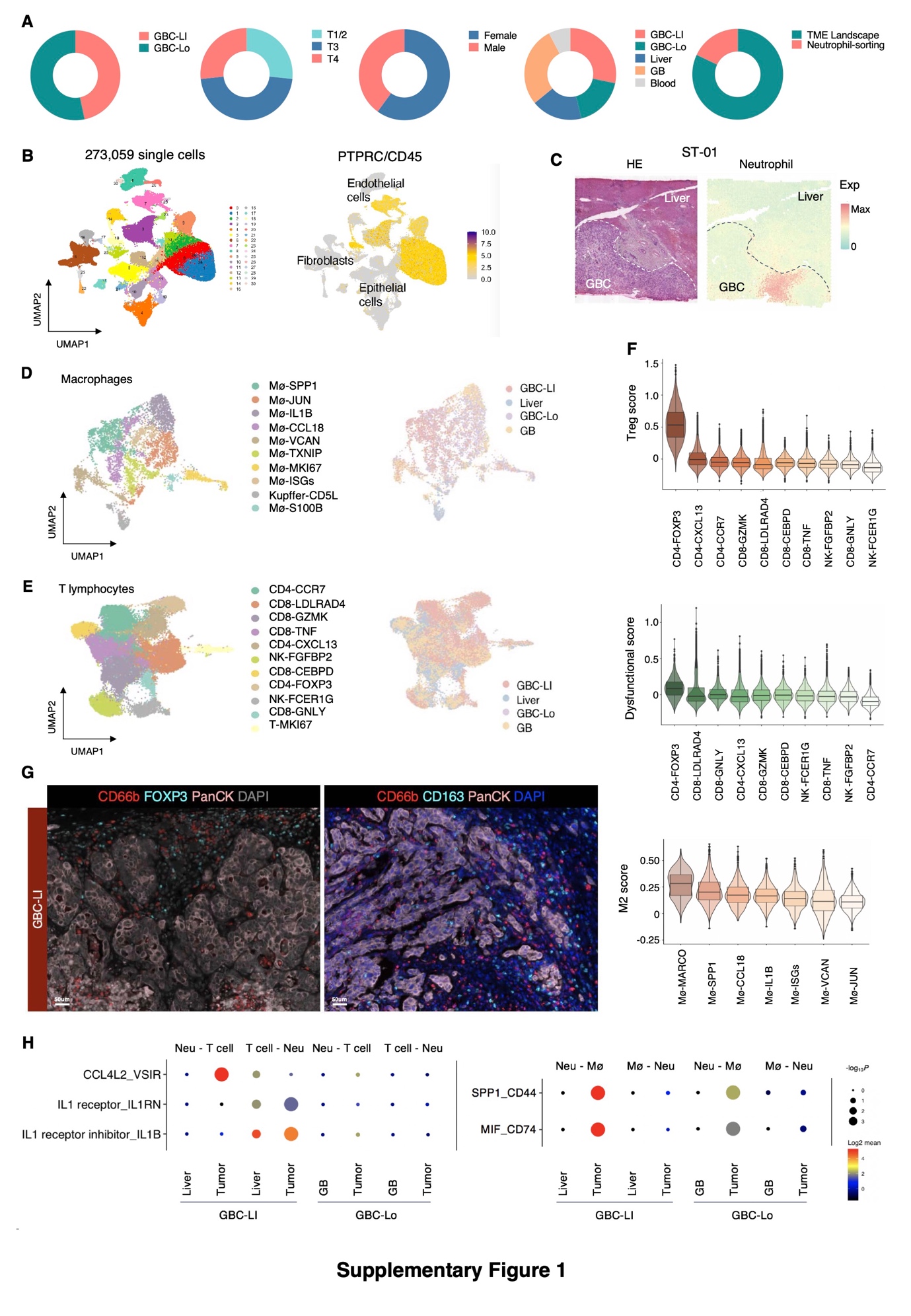
**

**Figure S1 | Characteristics of major immune components in GBC TME**

**(A)** Clinical parameters of patients (left) and samples (right) for scRNA analysis.

**(B)** UMAP of all single cells colored by cluster identities (left) and showing the expression PTPRC/CD45 (right).

**(C)** Hematoxylin-eosin (HE) staining of ST-01, and the expression of neutrophil signatures.

**(D** and **E)** UMAP of macrophages **(D)** and T lymphocytes (including T cells and NK cells) **(E)** labeled by cluster identity and sample origin.

**(F)** Violin plot showing the expression level of functional scores for T lymphocyte and macrophage clusters.

**(G)** Representative mIHC images of CD66b^+^ neutrophils, FOXP3^+^ Tregs (left), and CD163+ macrophages (right) on sections of GBC-LI.

**(H)** CellPhoneDB based analysis for ligand-receptor pairs between neutrophils and T cells (left) or macrophages (right) from different samples.


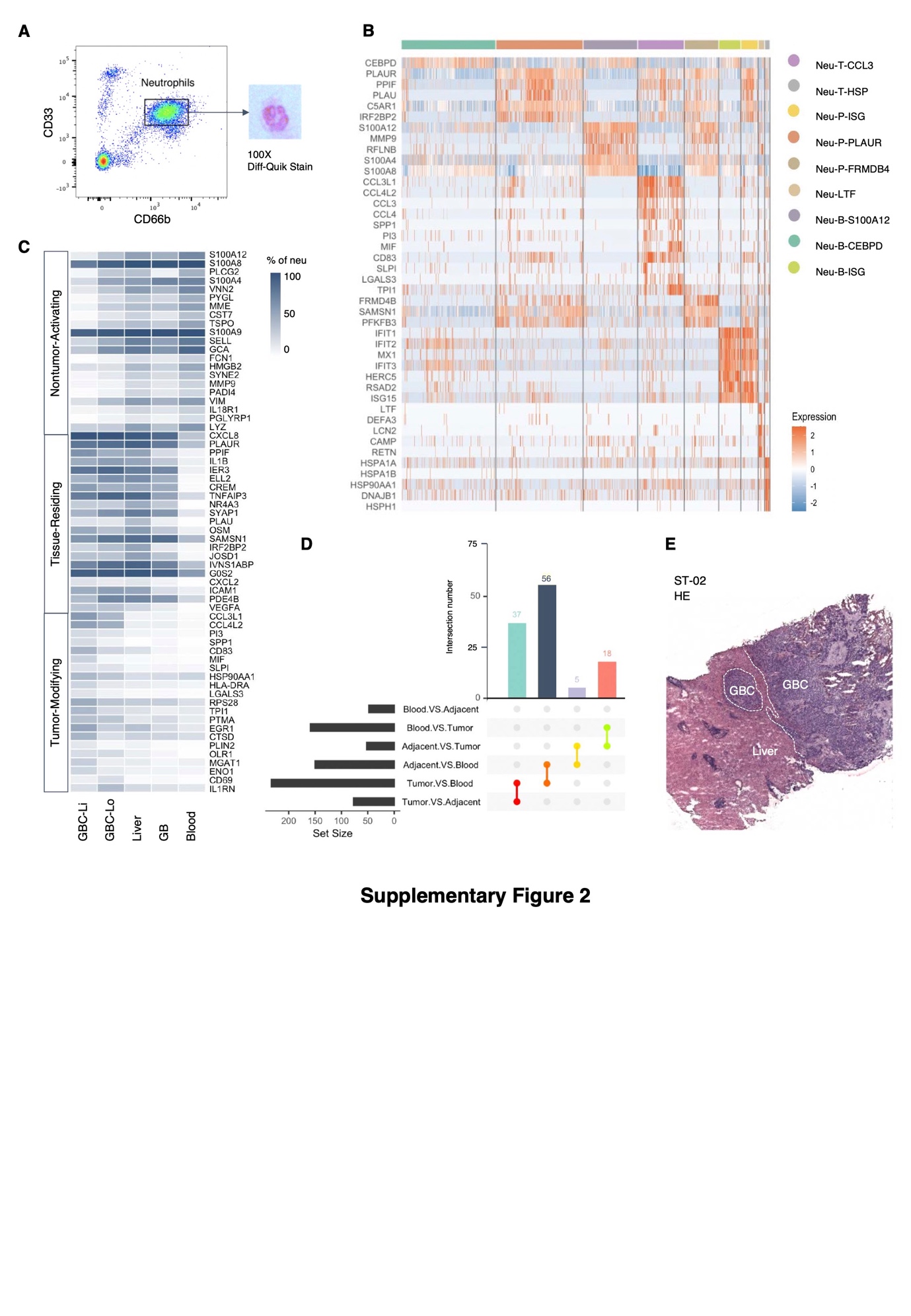


**Figure S2 | Neutrophil diversity as indicated by the context-dependent scores**

**(A)** Gating strategy (left) for FACS sorting and the representative image (right) of sorted neutrophils stained with Diff-Quik Stain.

**(B)** Heatmap showing the expression of specific genes in each neutrophil cluster.

**(C)** Heatmap showing the expression percentage of neutrophils from each sample type for genes in context-dependent scores.

**(D)** UpSet plot of differentially expressed genes in pairwise comparison for each tissue with the rest of the tissues.

**(E)** HE staining of ST-02. Dash lines in white draw the GBC outline.


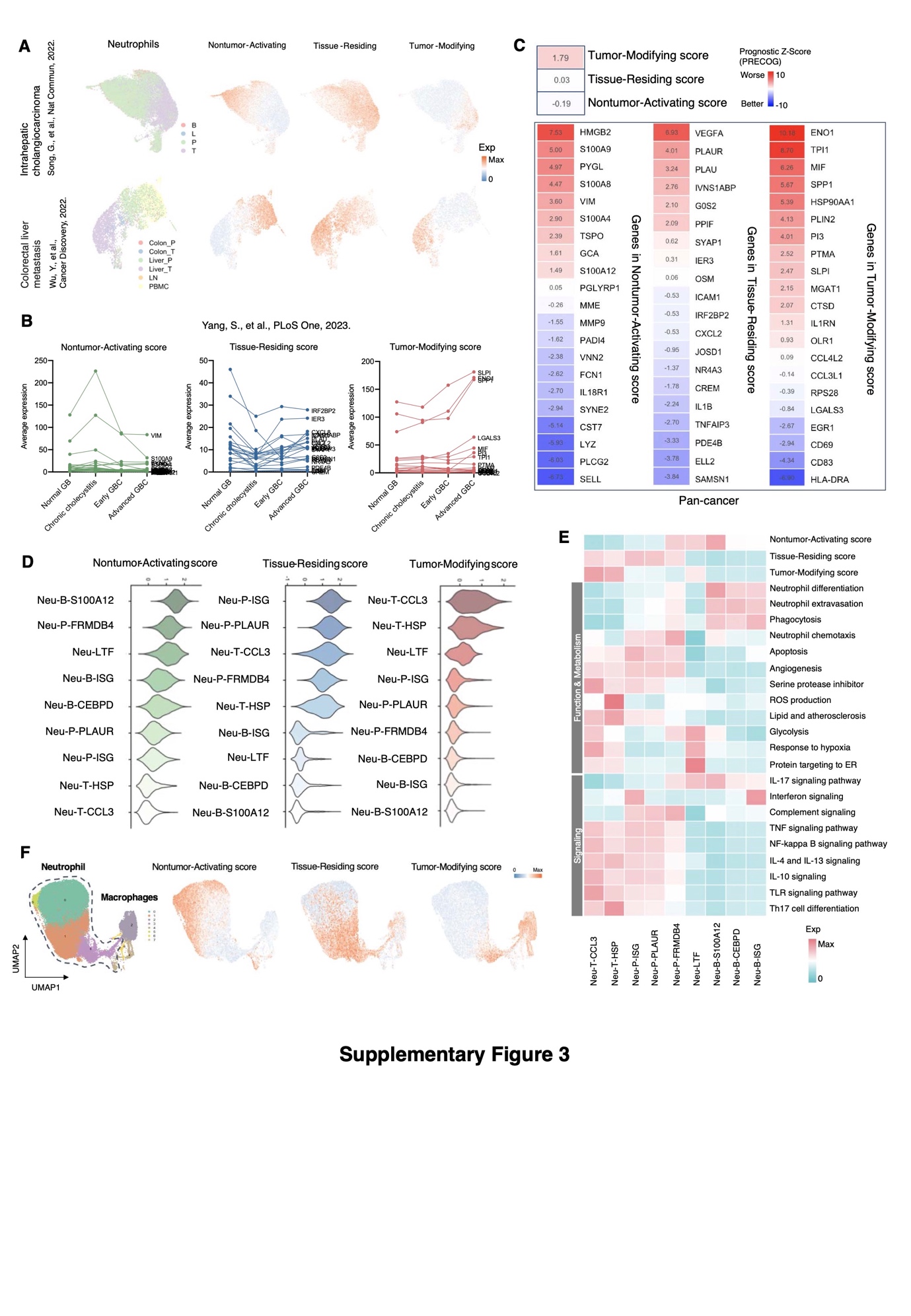


**Figure S3 | Prognostic and Biological significance of the context-dependent scores**

**(A)** Context-dependent scores for neutrophils in scRNA-seq data of intrahepatic cholangiocarcinoma (Song, G., et al., Nat Commun, 2022.) and colorectal cancer liver metastasis (Wu, Y., et al., Cancer Discovery, 2022.).

**(B)** Average expression of genes in context-dependent scores in different tissue samples from another GBC study (Yang, S., et al., PLoS One, 2023.)

**(C)** Prognostic z-scores for genes in the context-dependent scores. Negative and positive prognostic *Z*-scores respectively associate with favorable and adverse prognosis.

**(D)** Neutrophil clusters in order of descending expression level of each context-dependent score.

**(E)** Heatmap showing the expression of pathways enriched in each neutrophil cluster.

**(F)** UMAP of all myeloid cells (neutrophil and macrophage is annotated), colored by cluster identities and expression of the context-dependent scores.

**
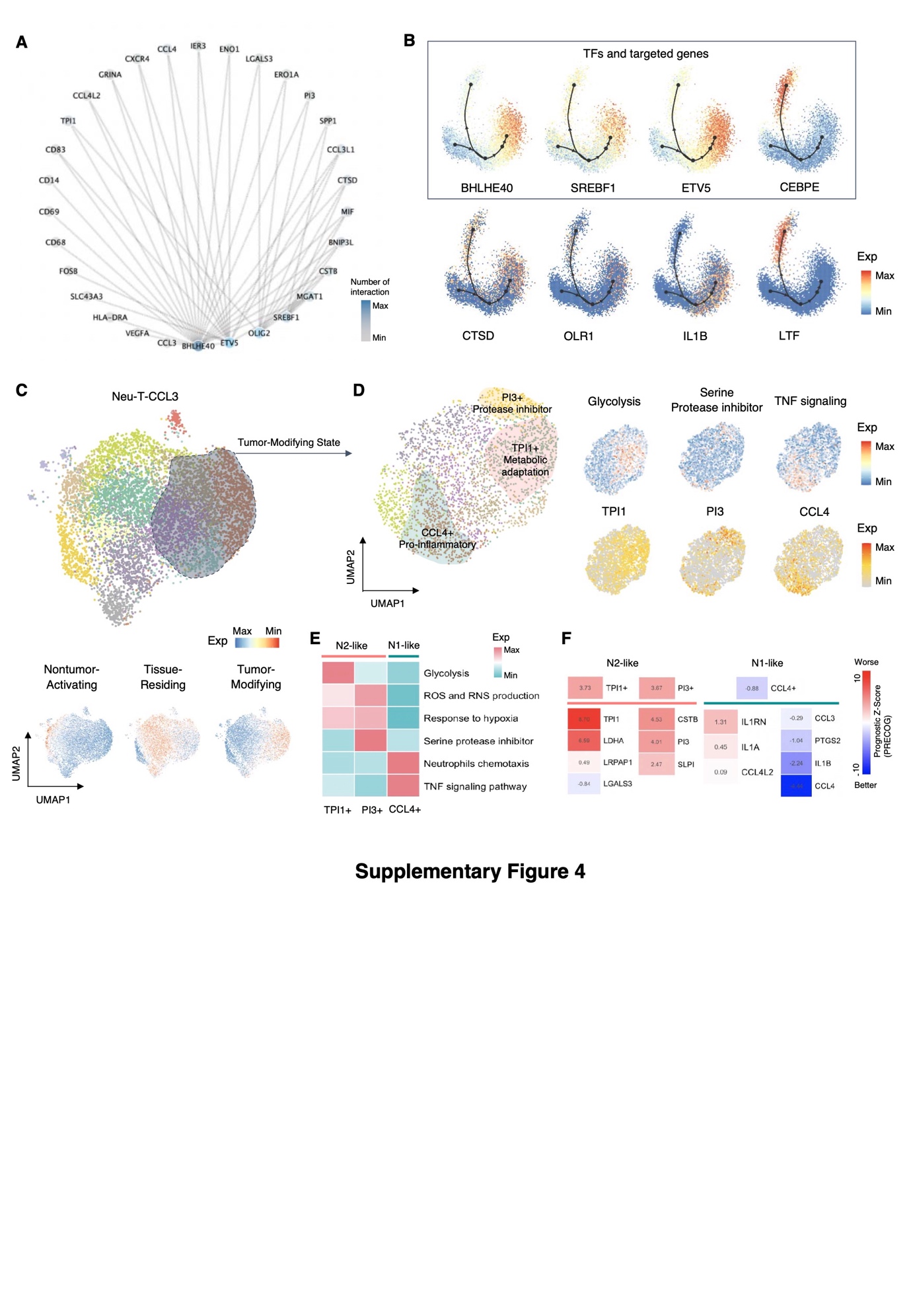
**

**Figure | S4 Transcriptional factors and the heterogeneity of tumor-neutrophils**

**(A)** Top transcriptional factors and their targeted genes predicted by SCENIC.

**(B)** Expression of top transcriptional factors and their targeted genes on slingshot trajectory of neutrophils.

**(C)** UMAP of the reclustered Neu-T-CCL3 and expression of the context-dependent scores, neutrophil clusters of typical Tumor-Modifying state were annotated by dash outline filled with shadow.

**(D)** UMAP of the reclustered neutrophils of Tumor-Modifying state in **C** and expression of representative pathways and genes.

**(E)** Heatmap showing the expression of pathways enriched in N1- and N2- like neutrophils.

**(F)** Prognostic z-scores for signature genes of N1- and N2- like neutrophils. Negative and positive prognostic *Z*-scores respectively associate with favorable and adverse prognosis.

**
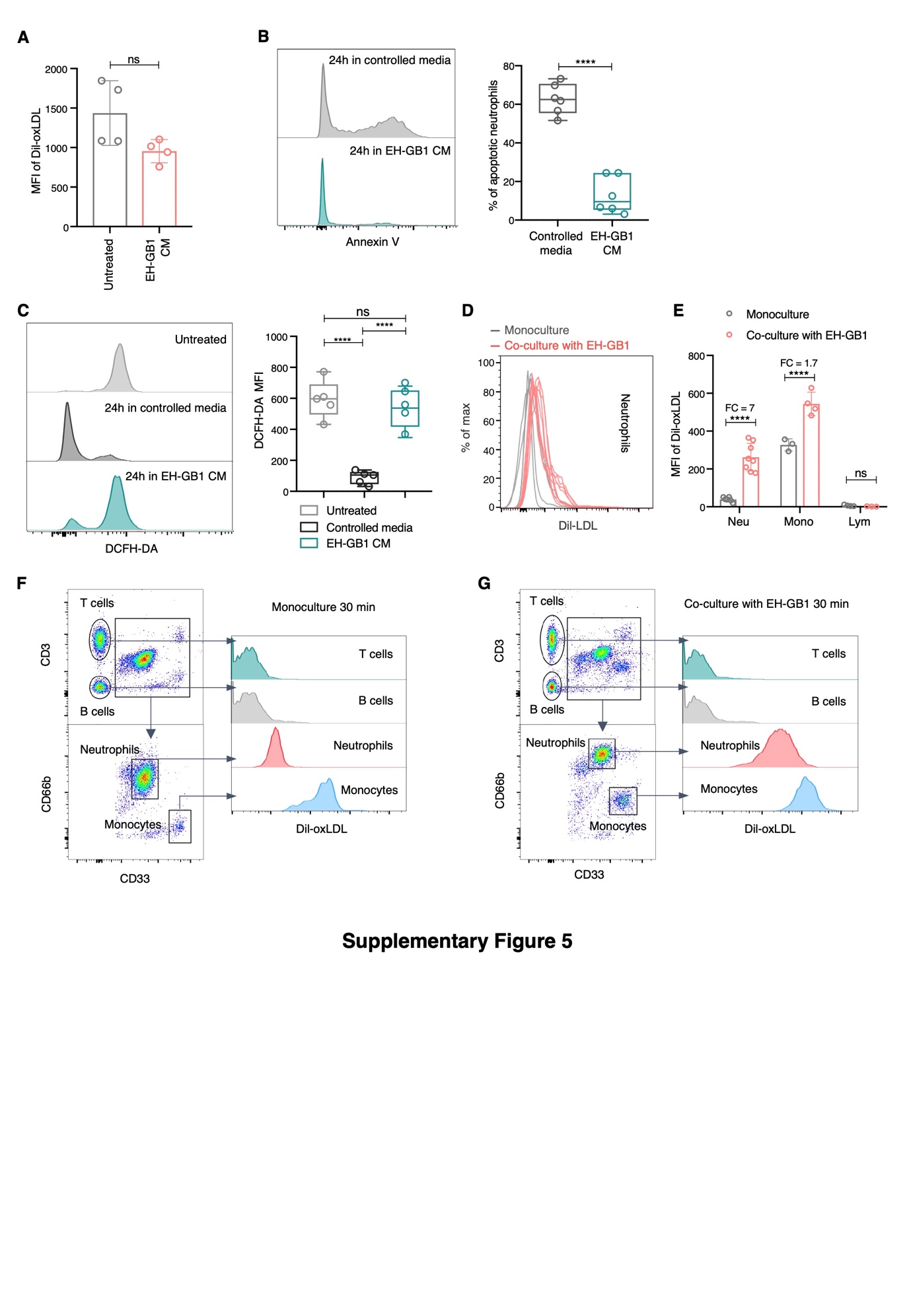
**

**Figure S5 | Cholesterol uptake of immune cells in response to GBC cells**

**(A)** OxLDL uptake of untreated neutrophils and neutrophils cultured in EH-GB1 conditioned media (CM). Comparison was performed using two-tailed unpaired t-test.

**(B** and **C)** Representative histogram and quantification of apoptosis percentage **(B)** and ROS production **(C)** for neutrophils cultured in controlled media or EH-GB1 CM for 24h as measured by flow cytometry. Comparison was performed using two-tailed unpaired t-test in **B**. Statistical analyses were performed using two-way ANOVA with Sidak's multiple comparisons test in **C**.

**(D)** Representative histogram of LDL uptake of neutrophils when monoculture and co-cultured with EH-GB1.

**(E)** Quantification of oxLDL uptake by neutrophils, monocytes, and lymphocytes when monoculture and co-cultured with EH-GB1. Two-way ANOVA with Sidak's multiple comparisons test.

**(F** and **G**) Gating strategy for T cells (CD33^-^ CD3^+^), B cells (CD33^-^ CD3^-^), monocytes (CD3^-^ CD33^high^ CD66b^-^), and neutrophils (CD3^-^ CD33^mid^ CD66b^+^), and their oxLDL uptake when monoculture **(F)** and co-cultured with EH-GB1 **(G)**.

Data are presented as mean with SD. *P < 0.05, **P < 0.01, ***P < 0.001, ****P < 0.0001.

**
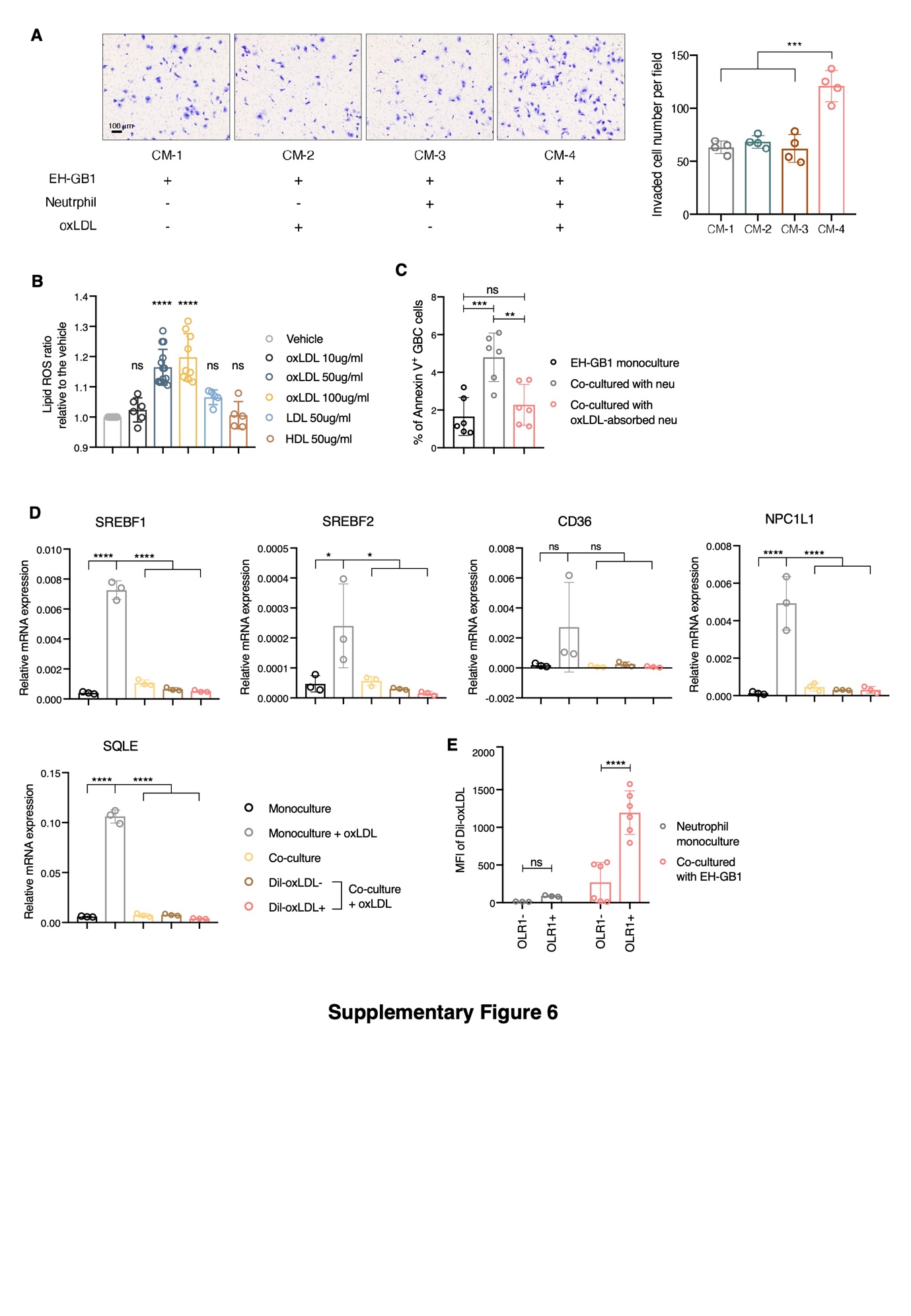
**

**Figure S6 | Alteration of neutrophils in response to oxLDL**

**(A)** Transwell invasion assay, representative images and quantification of the invaded GBC cells. Cells were counted in each 20x field-of-view image. Two-way ANOVA with Sidak's multiple comparisons test.

**(B)** Quantification of lipid ROS ratio in neutrophils coculture with EH-GB1 and supplied with LDL, HDL and oxLDL of different concentration. The lipid ROS and total ROS level as labeled by BODIPY 581/591 C11 were detected on FITC and PE via flow cytometry respectively, thus the lipid ROS ratio was calculated by BODIPY C11 MFI on FITC / BODIPY C11 MFI on PE. One-way ANOVA with Tukey’s multiple comparisons test.

**(C)** Quantification of apoptotic percentage of EG-GB1 cells when monoculture and co-cultured with neutrophils with or without oxLDL supplement as measured by flow cytometry. Statistical analyses were performed using one-way ANOVA with Bonferroni’s multiple comparisons test.

**(D)** Relative mRNA expression of cholesterol-related genes in neutrophils as measured by qRT-PCR, when monoculture or co-cultured with EH-GB1, with or without oxLDL supplement. One-way ANOVA with Tukey’s multiple comparisons test.

**(E)** Quantification of oxLDL uptake in neutrophils stratified by OLR1 expression after monoculture or co-cultured with EH-GB1 for 30 min as measured by flow cytometry. Two-way ANOVA with Sidak's multiple comparisons test.

Data are presented as mean with SD. *P < 0.05, **P < 0.01, ***P < 0.001, ****P < 0.0001.

**
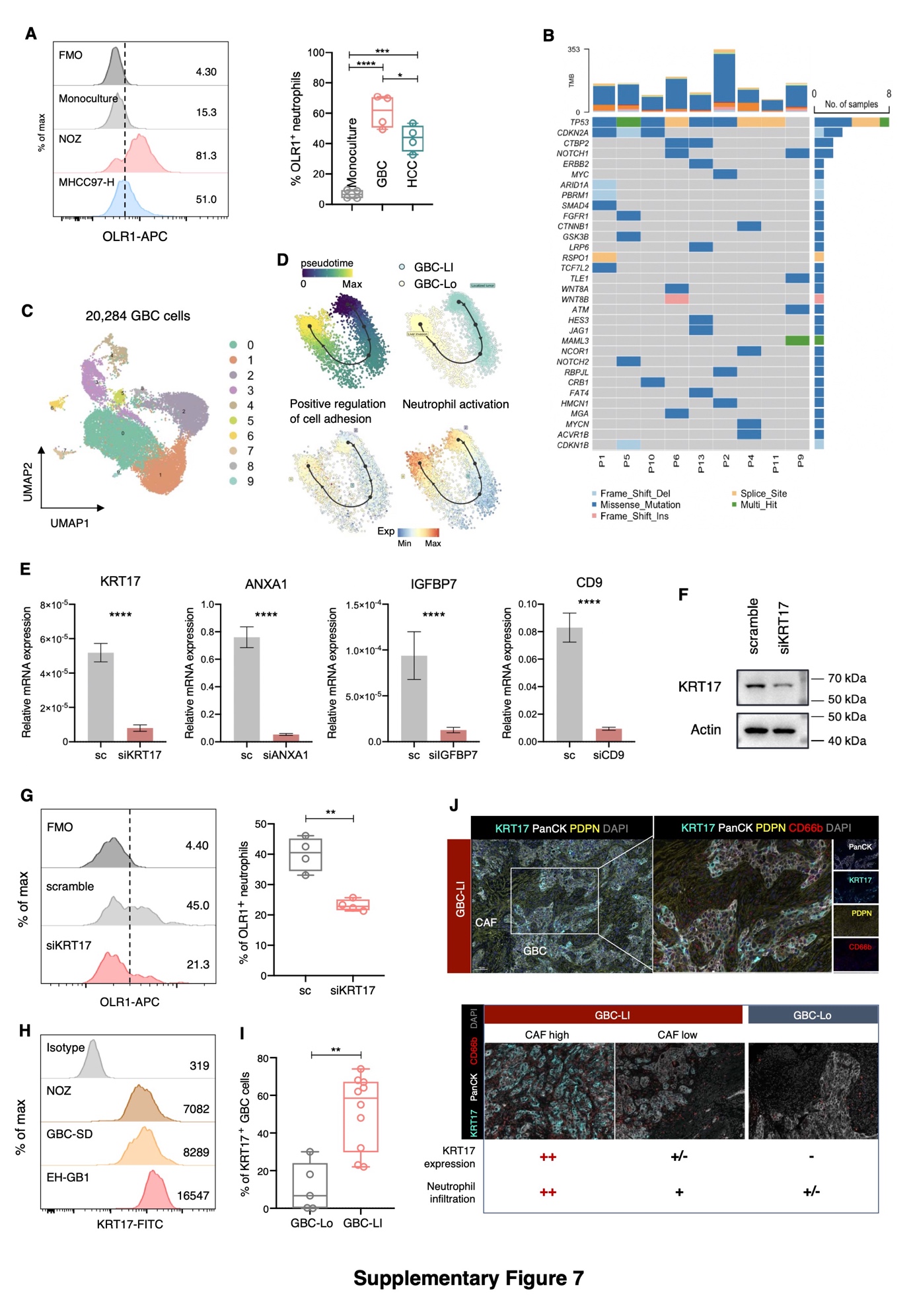
 Figure S7 | Characteristics of KRT17^+^ GBC cells**

**(A)** Neutrophils were co-cultured with different GBC and HCC cell lines. Representative histogram and quantification showing the percentage of OLR1+ neutrophils. One-way ANOVA with Tukey’s multiple comparisons test.

**(B)** Top histogram showing the TMB per sample; right histograms, frequency of mutation events; Bottom heatmap displaying the distribution of mutation events among patients.

**(C)** UMAP of GBC cells, colored by cluster identity.

**(D)** Slingshot trajectory analysis of GBC cells, colored by pseudotime, sample origin, and expression of the representative pathways.

**(E)** QRT-PCR validation of siRNA knockdown of KRT17, ANXA1, IGFBP7, and CD9. Comparison was performed using two-tailed unpaired t-test.

**(F)** Western blot validation of siRNA knockdown of KRT17.

**(G)** Representative histograms and quantifications showing the percentage of OLR1+ neutrophils after co-culture with EH-GB1 of siRNA knockdown of KRT17. Comparison was performed using two-tailed unpaired t-test.

**(H)** Histogram showing the KRT17 expression of different GBC cell lines.

**(I)** Percentage of KRT17+ cells in GBC cells from different tumor samples of GBC-Lo and GBC-LI based on the scRNA-seq data. Comparison was performed using two-tailed unpaired t-test.

**(J)** Top, representative mIHC images of KRT17, PanCK, PDPN, and CD66b staining on sections of GBC; bottom, the summary of the relation between the CAF infiltration and the distribution of KRT17+ GBC cells and neutrophils.


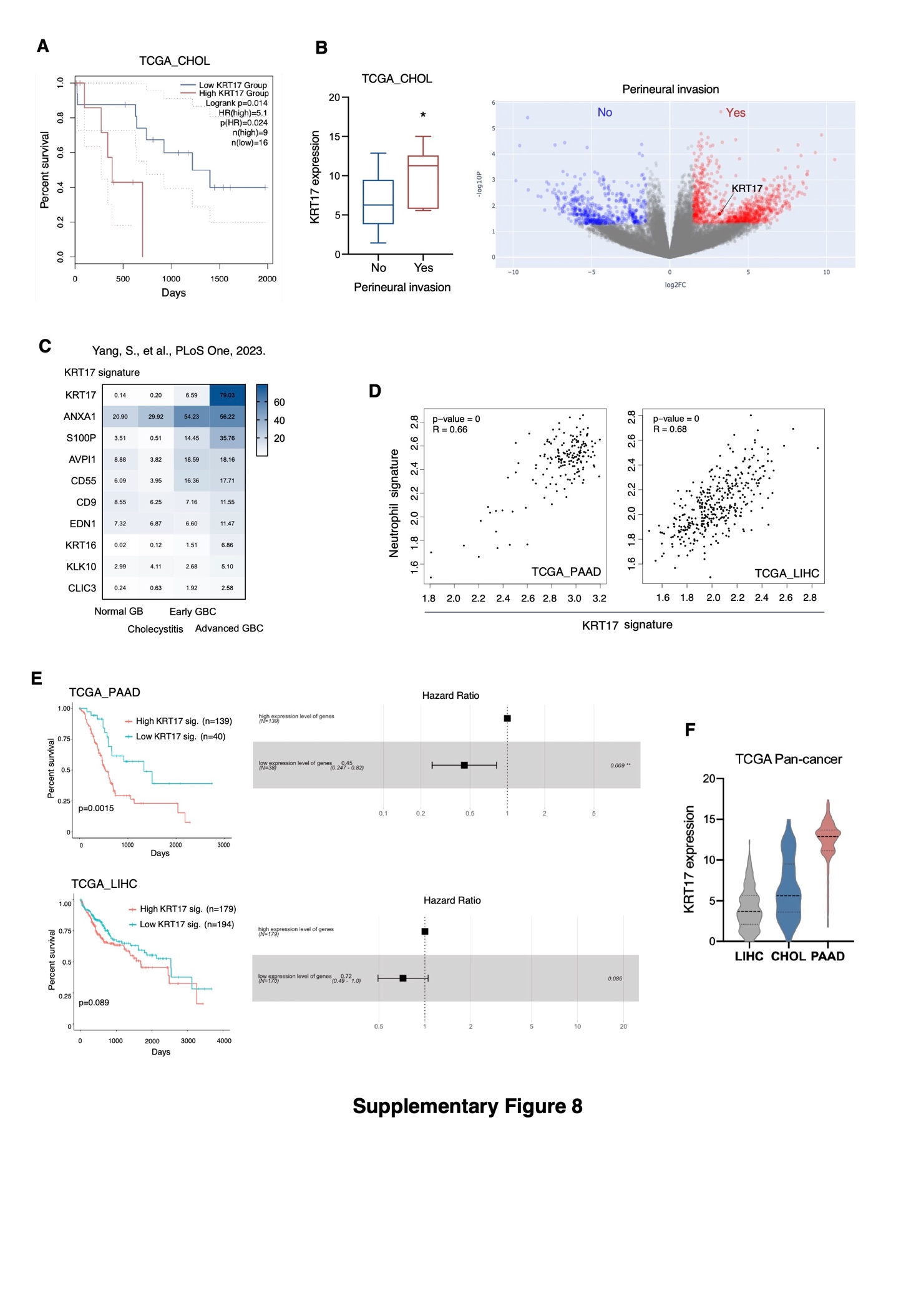


**Figure S8 | Prognostic significance of KRT17 signature**

**(A)** The Kaplan–Meier overall survival curves of bile duct cancer (TCGA_CHOL) stratified by KRT17 expression.

**(B)** Left, KRT17 expression in CHOL with or without perineural invasion; right, DEGs between CHOL with perineural invasion and without. Comparison was performed using two-tailed unpaired t-test.

**(C)** Heatmap showing the average expression of KRT17 signature genes in different tissue types from another GBC study (Yang, S., et al., PLoS One, 2023.).

**(D)** Scatter plot displaying the correlation between KRT17 and neutrophil signature in pancreatic cancer (TCGA_PAAD) and liver cancer (TCGA_LIHC).

**(E)** The Kaplan–Meier overall survival curves of PAAD and LIHC patients stratified by KRT17 signature expression. The ward.D2 method were used to perform unsupervised hierarchical clustering, and the emerging subgroups of patients were used for survival analysis.

**(F)** KRT17 expression in LIHC, CHOL, and PAAD from TCGA.

**Table S1.** Clinical characteristics of GBC patients from the scRNA-seq cohort

| Patient | Gender | Age | Pathology | T category | Stage | Type |
| --- | --- | --- | --- | --- | --- | --- |
| P1 | F | 65 | Adenocarcinoma | T3 | Locally advanced | GBC-Lo |
| P2 | F | 65 | Adenocarcinoma | T2a | Early | GBC-Lo |
| P4 | F | 76 | Adenosquamous | T3 | Locally advanced | GBC-Lo |
| P5 | M | 67 | Undifferentiated | T3 | Locally advanced | GBC-Lo |
| P6 | M | 71 | Adenocarcinoma | T4 | Metastatic/Invasive | GBC-LI |
| P7 | F | 70 | Adenocarcinoma | T3 | Metastatic/Invasive | GBC-LI |
| P8 | M | 63 | Adenocarcinoma | T1b | Early | GBC-Lo |
| P9 | F | 63 | Adenocarcinoma | T4 | Metastatic/Invasive | GBC-LI |
| P10 | M | 62 | Adenocarcinoma | T3 | Metastatic/Invasive | GBC-LI |
| P11 | F | 63 | Adenocarcinoma | T4 | Metastatic/Invasive | GBC-LI |
| P12 | F | 65 | Adenocarcinoma | T1b | Early | GBC-Lo |
| P16 | M | 59 | Adenocarcinoma | T2a | Early | GBC-Lo |
| P13 | M | 62 | Adenocarcinoma | T4 | Metastatic/Invasive | GBC-LI |
| P14 | F | 67 | Adenocarcinoma | T3 | Metastatic/Invasive | GBC-LI |
| P15 | F | 69 | Adenocarcinoma | T3 | Locally advanced | GBC-Lo |

**Table S2.** Summary of analytical methods

| Software | Source |
| --- | --- |
| Linux | https://www.linux.org/ |
| Anaconda | https://www.anaconda.com/ |
| Python | https://www.python.org/ |
| R | https://www.r-project.org/ |
| Rstudio | https://www.rstudio.com/ |
| Cell Ranger | https://support.10xgenomics.com/ |
| Seurat | https://github.com/satijalab/seurat |
| harmony | https://github.com/immunogenomics/harmony |
| InferCNV | https://github.com/broadinstitute/inferCNV |
| pySCENIC | https://github.com/aertslab/pySCENIC |
| dyno | https://github.com/dynverse/dyno |
| PAGA | https://github.com/theislab/paga |
| Monocle2 | https://github.com/cole-trapnell-lab/monocle2-rge-paper |
| clusterProfiler | https://github.com/YuLab-SMU/clusterProfiler |
| dplyr | https://github.com/tidyverse/dplyr |
| ggplot2 | https://github.com/tidyverse/ggplot2 |
| SingleR | https://github.com/dviraran/SingleR |
| DoubleFinder | https://github.com/chris-mcginnis-ucsf/DoubletFinder |
| DoubletDetection | https://github.com/JonathanShor/DoubletDetection |
| Scrublet | https://github.com/AllonKleinLab/scrublet |
| CSOmap | https://github.com/zhongguojie1998/CSOmap |
| CellphoneDB | https://github.com/Teichlab/cellphonedb |
| BayesSpace | https://github.com/edward130603/BayesSpace/ |
| Trim Galore | https://github.com/FelixKrueger/TrimGalore |
| BWA-MEM | https://github.com/lh3/bwa |
| STAR | https://github.com/alexdobin/STAR |
| Samtools | https://github.com/samtools |
